# Increased triacylglyceride and ceramide levels are key for MERS-CoV replication

**DOI:** 10.1128/msphere.00523-25

**Published:** 2026-01-15

**Authors:** Hugh D. Mitchell, Jennifer Kyle, Kristin Engbrecht, Madelyn Berger, Kristie L. Oxford, Amy C. Sims

**Affiliations:** 1 Earth and Biological Sciences Directorate, Pacific Northwest National Laboratory 6865, Richland, Washington, USA; 2 National Security Directorate, Pacific Northwest National Laboratory 6865, Richland, Washington, USA; 3 Affiliate Professor, Department of Comparative Medicine, University of Washington 312755, Seattle, Washington, USA; Instituto de Biotecnologia/UNAM, Cuernavaca, Morelos, Mexico

**Keywords:** MERS-CoV, infection phenotypes, lipid metabolism, proteomics, ACSL3, lipidomics

## Abstract

Emerging viruses remain a threat to human health; however, many aspects of their infection cycle are still poorly understood. Host lipid structures and abundances are observed to be significantly altered during infection, and the mechanisms regulating lipid synthesis and modification remain largely unknown. In this work, we analyzed a large multi-omic data set from three Middle East respiratory syndrome coronavirus (MERS-CoV)-infected primary human lung cell types, all derived from three distinct donors to investigate the changes in lipid species during infection. Analysis of lipidomics data identified perturbations of various lipid classes, and we hypothesized and confirmed that MERS-CoV infection orchestrates an increase in ceramide via sphingomyelinase pathways required for infection. We also identified a minor subset of proteins with lipid-related functions with increased differential expression among a striking majority of lipid-related proteins with decreased differential expression. The most prominent of these is ACSL3, a long-chain acyl-CoA synthetase that is key for the synthesis of triacylglycerides and is associated with lipid droplet formation, an established feature of coronavirus-infected cells. Accordingly, the inhibition of acyl-CoA synthetase activity reduced MERS-CoV replication. These results suggest a model wherein coronaviruses perturb overall cellular metabolism to shift resources to the production of ceramides and triacylglycerides, particularly through acyl-CoA synthetase activity. Our findings suggest a strategy for targeting CoV replication through the inhibition of specific subsets of lipid metabolism.

**IMPORTANCE:**

Combating emerging viral threats requires an in-depth understanding of how the virus commandeers host resources to facilitate replication. Viral particles are comprised of protein and lipids; hence, the synthesis of both is critical for virus spread. Our studies have demonstrated that the synthesis of two lipid species, ceramides and triacylglycerides, is essential for Middle East respiratory syndrome coronavirus replication and that virus replication is impaired if these synthetic pathways are blocked. These results suggest a model wherein coronaviruses perturb overall cellular metabolism to shift resources to the production of ceramides and triacylglycerides. Our findings suggest a strategy for targeting coronavirus replication through the inhibition of specific subsets of lipid metabolism.

## INTRODUCTION

The threat of novel emerging virus outbreaks continues to increase into the third decade of the 21st century, underscoring the urgent need to identify therapeutic and prophylactic antivirals. Since 2002, the emergence of three highly pathogenic human coronaviruses has significantly impeded global trade and travel multiple times, resulting in major economic losses and millions of untimely deaths. In 2012, Middle East respiratory syndrome coronavirus (MERS-CoV), the causative agent of MERS, was identified, and to date, it has caused >2,600 human infections with a mortality rate of ~35% (1). The zoonotic reservoir for MERS-CoV is the Arabian Peninsula dromedary camel. Camel-to-human transmission continues due to the cultural importance of camels in the Middle East, where they are kept as pets or used for sport, meat, and transportation (2). Interestingly, MERS-CoV-like strains are also enzootic in African dromedary camels, but these strains do not readily transmit to humans (2). Ongoing investigations are exploring the phylogeny of the Arabian and African MERS-CoV lineages to identify the determinants of transmissibility; however, given the high mortality rate of documented MERS-CoV infection in humans and that >70% of African dromedaries are infected with MERS-CoV-like strains, the risk for a global MERS-CoV pandemic increases (2).

Viral entry, replication, virion assembly, and egress depend on interactions with host cell membranes and a variety of lipid species. To avoid host defense mechanisms and shunt resources to sites of viral replication, positive-sense single-stranded viral RNA synthesis occurs in “replication factories” associated with invaginated intracellular membranes (3, 4). Coronavirus infection induces the formation of double membrane vesicles that can sequester and protect viral RNA species from host detection and allow close association of the viral nonstructural and host proteins required for RNA synthesis (5). Although it has been well demonstrated that intracellular membranes are key for viral replication, including MERS-CoV replication (5–7), the contribution of individual lipid species and proteins with lipid-associated functions is not well understood.

**Fig 1:**
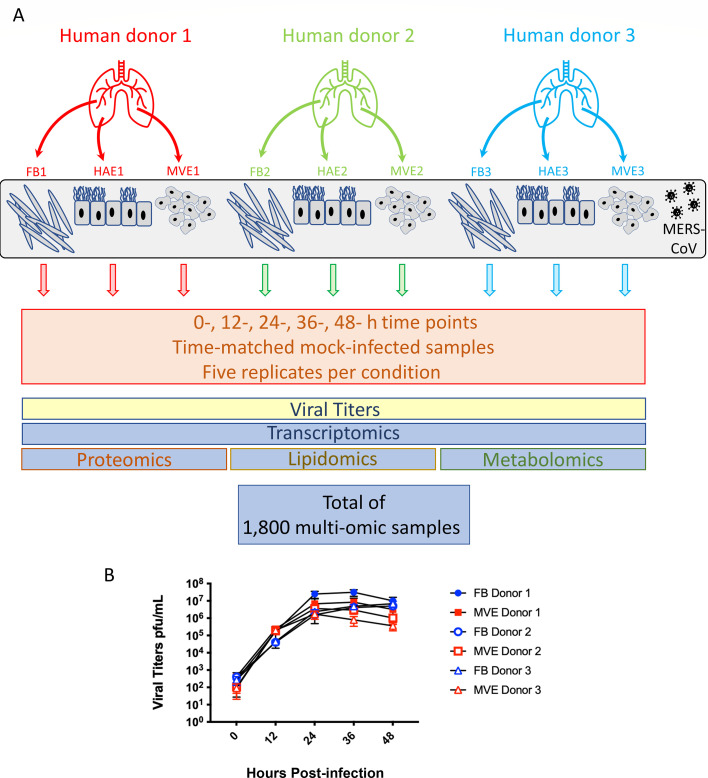
Schematic of MERS-CoV infection of primary human lung cells, sample collection strategy and levels of MERS-CoV replication. (**A**) Schematic of MERS-CoV infection of primary human lung cells and sample collection strategy. Three cell types were isolated from human donor lung tissue (fibroblasts, airway epithelium, and microvascular epithelium; each cell type from the same three donors) and cultured. Cultures were exposed to MERS-CoV and harvested at five times following infection: 0, 12, 24, 36, and 48 h. In parallel, mock-infected cultures were incubated and harvested at the same times following infection. Five replicates are prepared for each condition (donor, tissue type, and time point). Viral titers were determined for all infected samples, and two sets of samples were harvested for either transcriptomics or using a separation protocol for isolating proteomic, lipidomic, and metabolomic fractions from a single sample and subsequent mass spectrometry. (**B**) At each time period post-infection, samples were taken to assess MERS-CoV growth kinetics in primary human lung microvascular endothelial cells and fibroblasts. Samples were assayed using plaque assays in Vero 81 cells, and the results are shown as plaque-forming units per milliliter. The graph contains data from all three donors for microvascular endothelial cells and fibroblasts, as these cell types are the focus of the current study. Similar titers were obtained for MERS-CoV-infected human airway epithelial cell cultures derived from the lungs of the same donors (9). FB, fibroblast; MVE, microvascular endothelial cells; HAE, human airway epithelial cell cultures; and MERS-CoV, Middle East respiratory syndrome coronavirus.

Defining host cell functions required for productive viral replication and transmission using systems biology approaches can identify new targets for treatment options to prevent virus spread and/or lessen disease severity. Unlike severe acute respiratory syndrome coronaviruses (SARS-CoV and SARS-CoV-2), MERS-CoV infection has an expanded tropism within the human lung and can infect fibroblasts and endothelial cells in addition to epithelial cells (8, 9). To explore MERS-CoV infection in different cell types within the human lung, we previously collected and analyzed a large multi-omics data set over a 48-h time course and found unique cell type-specific responses of total RNA, protein, lipids, and metabolites derived from MERS-CoV-infected primary human lung cells obtained from three tissue donors (9) (Fig. 1). Data sets derived from infected immortalized human lung epithelial cells highlighted key changes to host transcript and protein expression, and frequently, these data overlap with representative responses in primary cells (9, 10); however, immortalized cells can have altered cellular functions. Thus, the use of primary cells offers the advantage of examining responses from individual cell types derived from human lung tissue and is more representative of responses in the human lung.

MERS-CoV primary human lung cell data sets have been analyzed using a variety of approaches to define cellular pathways and gene networks altered by MERS-CoV infection (9). These analyses linked the activation of the unfolded protein response (UPR) to MERS-CoV infection-induced apoptosis in infected primary human lung microvascular endothelial cells, demonstrating that inhibition of the UPR reduced MERS-CoV replication in the primary cells and reduced disease severity in a rodent model of MERS-CoV pathogenesis (9). The UPR-linked apoptosis study relied primarily on transcriptomic and proteomic data sets; however, it was also noted that MERS-CoV infection altered the expression of a variety of genes and proteins associated with lipid metabolism in all three cell types—trends that were conserved across all three donors. As lipids play important roles in mediating cellular structure and integrity, intra- and extra-cellular signaling, immune responses, and/or therapeutics/prophylactics (11–13), improving current systems biology approaches to include lipidomics can greatly enhance our understanding of the contributions of lipid subclasses and individual species during virus infection.

In the current study, we compared the functional enrichment results from transcript and protein and lipid mass spectrometry data across major cell types in lung tissues with the expression patterns of proteins with known lipid-associated functions to understand the role of lipid metabolism in CoV replication. MERS-CoV infection caused differential expression of a variety of ceramide, sphingomyelin (SM), and triacylglyceride (TG) lipid species. We identified acyl-CoA synthetase 3 (ACSL3) as among the few proteins with lipid-associated functions that had increased differential expression across cell types, and we used various inhibitors to determine how altering the phenotypes of lipid synthesis pathways affected MERS-CoV infection. ACSL3 inhibitor-based verification studies verified the role of lipid synthesis as a key element for MERS-CoV replication.

## RESULTS

### MERS-CoV infection induces differential expression of transcripts and proteins with lipid-associated functions

**Fig 2:**
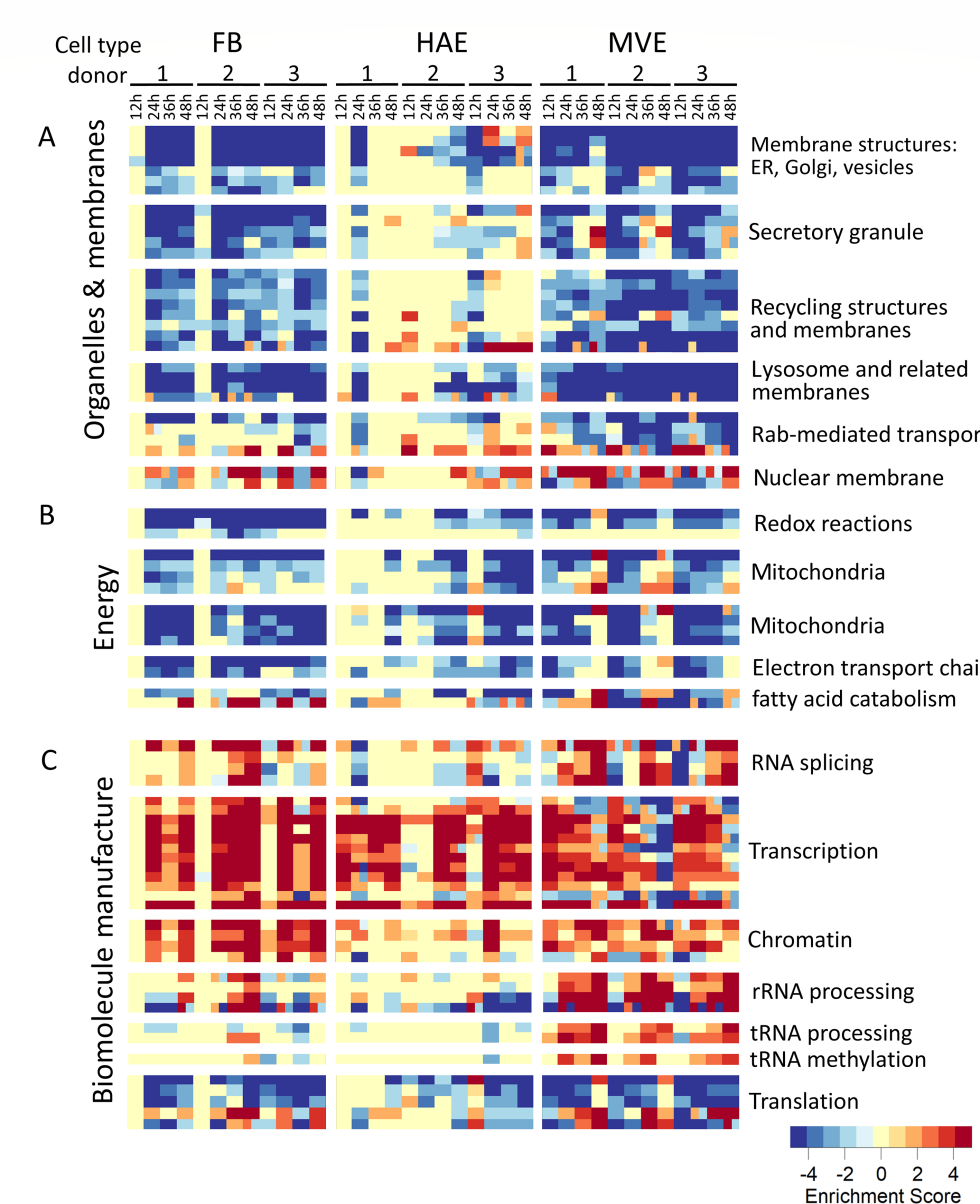
Genes with lipid-associated functions or functions within lipid-related pathways have reduced levels of transcription following MERS-CoV infection. Functional enrichment analysis was performed for each condition based on differential gene expression compared with time-matched mocks, with results for individual donors performed separately. Color intensities represent −log10 of the *P* values of the significance of each enrichment test, after a multiple testing correction. Log *P*-values of pathways found to be enriched among downregulated genes were given a negative sign, whereas *P*-values from pathways enriched among upregulated genes were left positive. After performing hierarchical clustering, functions/pathways were grouped manually according to biological similarity as shown, with names assigned to each group manually according to overall function of pathways to each group. Downregulated pathways are depicted in blue tones, whereas red tones indicate upregulated pathways. (**A**) Cluster of pathways related to organelles and membranes. (**B**) Cluster of pathways related to energy. (**C**) Cluster of pathways related to biomolecule manufacture. FB, fibroblast; MVE, microvascular endothelial cells; and HAE, human airway epithelial cell cultures.

To determine how human coronavirus infection alters lipid-associated transcripts and proteins, we investigated MERS-CoV infection of three distinct primary human lung cell types derived from three tissue donors: human airway epithelial (HAE), microvascular endothelial (MVE), and fibroblasts (FB) as previously described (9). The experimental design is described in detail in Fig. 1. Briefly, HAE, MVE, and FB were infected with wild-type MERS-CoV at a multiplicity of infection (MOI) of 5, and the samples were collected at the indicated times post-infection. Parallel sets of samples were harvested in Trizol for transcriptomics analysis or in 2:1 chloroform methanol to isolate proteins, metabolites, and lipids per replicate sample (14–16). In addition, media from infected wells of MVE and FB and apical washes from HAE were collected to determine viral replication kinetics. In each cell type and donor, replication kinetics were similar over time with peak titers occurring by 24 h post-infection reaching ~10e6–10e7 plaque-forming units (pfu)/mL (Fig. 1B). Functional enrichment analysis focused on differential expression of transcripts (Fig. 2) above/below the mock levels following MERS-CoV infection, so that the direction of change between time points, donors, cell types, and omics platforms could be compared. In our previous study, we applied a threshold at the least significant result of the three donors for each Gene Ontology (GO) term (9). This highly conservative approach only revealed results common to all three donors, so that the resulting heatmap was somewhat sparse. For the current study, we retained results that appeared in any donor to capture a more complete picture of the infection response. To reduce the complexity of the various pathways that appear in Fig. 2, we have replaced the individual pathway names with group names that are manual summarizations of each indicated cluster and additionally grouped the clusters into collective super-clusters (“organelles and membranes,” “energy,” and “biomolecule manufacture”) (Fig. 2A through C). File S1 includes all original GO pathway names, as well as the summarized names present in the figure.

One striking response apparent from the transcriptome data were the widespread downregulation of terms related to intracellular membranes in FB and MVE (Fig. 2A). Previous work hinted at a mixed response of lipid-related transcripts but did not survey pathway level enrichment of lipid-/membrane-related activity (9, 17). In particular, we noted the downregulation of endoplasmic reticulum (ER)-related terms (encompassed in the top-most block of Fig. 2), since the ER is the main area of lipid synthesis. Although these results did not definitively demonstrate the downregulation of lipids *per se*, they made us speculate whether a general downregulation of lipid-related processes occurred during infection; this would suggest MERS-CoV-infected cells reduced lipid-related transcription to restrict lipid resources. This is interesting because lipids are a fundamental structural component of CoV virions and are required for the extensive rearrangement of intracellular membranes during CoV infection; therefore, lipid species with increased expression are likely important for viral replication and/or virion release (5, 7, 18). In contrast, in all three cell types and across the infection time course, gene expression was increased for cellular mRNA species that mediate transcription, maintenance of chromatin structures, and tRNA processing and methylation, demonstrating that not all transcription was decreased during CoV infection (Fig. 2C). Interestingly, transcripts that mediate cellular translation also had decreased expression following MERS-CoV infection, which may be indicative of conserving cellular resources to limit damage caused by infection (Fig. 2C).

### MERS-CoV infection decreases the expression of lipid-associated genes and proteins related to lysosomal-associated, lipid-associated transcription factor responses

MERS-CoV infection resulted in decreased expression of transcripts for many intracellular membrane and organelle-related gene sets in all three primary human lung cell types, especially MERS-CoV-infected FB and MVE (Fig. 2A). As a result, we were interested in further investigating the role of lipid-related genes in MERS-CoV infection. With this goal in mind, we plotted the overall trends of differential expression for lipid-related transcripts, using three large classes of lipid-related gene sets: lysosome-related genes, sterol regulatory element-binding protein (SREBP)-responsive genes, and transcription factor EB (TFEB)-responsive genes (see Methods). The predominant trend for genes classified as lysosomal-associated or SREBP- or TFEB-responsive was decreased expression (Fig. S1A through C, left panels, with numeric values provided in File S2). These trends for lysosomal-associated genes supported a previously published transcriptomics study where MERS-CoV NSP1 protein was overexpressed in *in vitro* and *in vivo* models to explore MERS-CoV NSP1’s role in autophagy (19). The authors demonstrated that NSP1’s endonuclease function contributed to the decrease in lysosomal-related transcripts (19). In contrast to these lipid-associated gene sets, the remainder of transcripts had relatively equal distributions between increased and decreased expression (Fig. S1A through C right panels), suggesting that the decrease in lipid-related gene transcription in MERS-CoV-infected cells was not due to widespread repression of transcription. In MERS-CoV-infected primary human lung cells, differential expression of proteins with lysosome-related functions or whose gene expression was regulated by SREBP or TFEB followed similar patterns to the transcriptomic data (Fig. S2A through C left panels). Globally, protein expression was reduced when compared with mock-infected samples, particularly in MERS-CoV-infected MVE (Fig. S2A through C right panels).

To compare our analysis of transcript expression from individual primary human lung cell types to transcriptomic results from an intact lung *in vivo*, we also examined the directional change of lipid-related transcripts from a data set derived from whole mouse lungs either mock-infected or infected with mouse-adapted MERS-CoV (GEO ID GSE108594; overall data set publication [20]). Lipid-related mouse genes were identified using the LIPID MAPS Proteome Database (21) (Fig. S3 left panel). As seen in the human lung primary cell data, changes in lipid-related murine genes were also overwhelmingly downregulated, whereas gene expression at large did not show a downward bias (Fig. S3 right panel).

**Fig 3:**
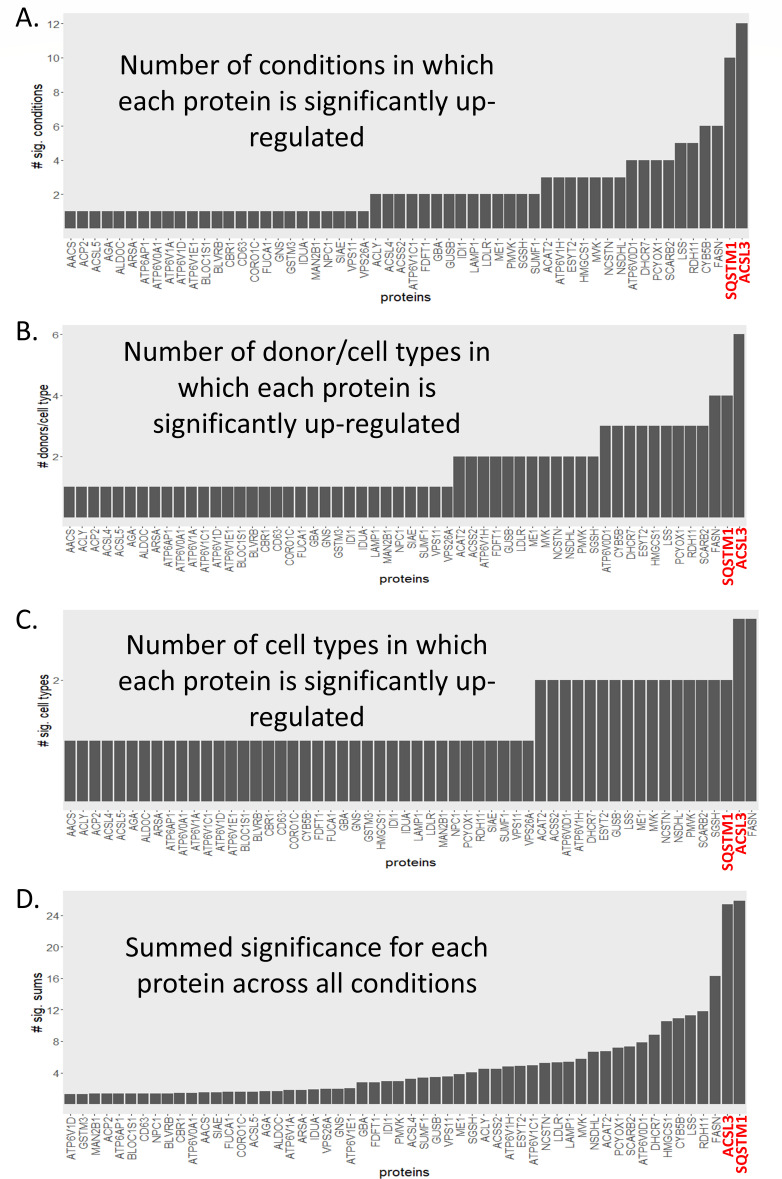
Ranking lipid-associated proteins according to frequency of significant upregulation following MERS-CoV infection in primary human lung cell types. (**A**) Frequency of lipid-associated protein upregulation among individual conditions (donor/cell type/time, maximum = 36 possible conditions), One is added to each protein’s score if it is significantly (adjusted *P*-value for the fold change < 0.05) upregulated in at least one condition. (**B**) Frequency of upregulation among donor/cell types, that is, for each donor/cell type combination, One is added to each protein’s score if it is significantly upregulated in at least one time point. (**C**) Frequency of up-regulation among cell types, that is one is added to each protein’s score if it is significantly up-regulated in at least one time point in at least one donor. (**D**) Summed significance for each lipid-associated protein across all conditions. The sum of all −log10 *P*-values of significance of upregulated lipid-associated proteins across all infection conditions is represented. Proteins of particular interest are highlighted in red text. AACS, Acetoacetyl-CoA synthetase; ACP2, Lysosomal acid phosphatase; ACSL5, Long-chain-fatty-acid-CoA ligase 5; AGA, N(4)-(beta-N-acetylglucosaminyl)-L-asparaginase; ALDOC, Fructose-bisphosphate aldolase C; ARSA, Arsenical pump-driving ATPase; ATP6AP1, V-type proton ATPase subunit S1; ATP6V0A1, V-type proton ATPase 116 kDa subunit a 1; ATP6V1A, V-type proton ATPase catalytic subunit A; ATP6V1D, V-type proton ATPase subunit D; ATP6V1E1, V-type proton ATPase subunit E 1; BLOC1S1, Biogenesis of lysosome-related organelles complex 1 subunit 1; BLVRB, Flavin reductase; CBR1, Carbonyl reductase [NADPH] 1; CD63, CD63 antigen; CORO1C, Coronin-1C; FUCA1, Tissue alpha-L-fucosidase; GNS, N-acetylglucosamine-6-sulfatase; GSTM3, Glutathione S-transferase Mu 3; IDUA, Alpha-L-iduronidase; MAN2B1, Lysosomal alpha-mannosidase; NPC1, NPC intracellular cholesterol transporter 1; SIAE, Sialate O-acetylesterase; VPS11, Vacuolar protein sorting-associated protein 11 homolog; VPS26A, Vacuolar protein sorting-associated protein 26A; ACLY, ATP-citrate synthase; ACSL4, Long-chain-fatty-acid-CoA ligase 4; ACSS2, Acetyl-coenzyme A synthetase, cytoplasmic; ATP6V1C1, V-type proton ATPase subunit C 1; FDFT1, Squalene synthase; GBA, Lysosomal acid glucosylceramidase; GUSB, Beta-glucuronidase; IDI1, Isopentenyl-diphosphate Delta-isomerase 1; LAMP1, Lysosome-associated membrane glycoprotein 1; LDLR, Low-density lipoprotein receptor; ME1, NADP-dependent malic enzyme; PMVK, Phosphomevalonate kinase; SGSH, N-sulphoglucosamine sulphohydrolase; SUMF1, Formylglycine-generating enzyme; ACAT2, Sterol O-acyltransferase 2; ATP6V1H, V-type proton ATPase subunit H; ESYT2, Extended synaptotagmin-2; HMGCS1, Hydroxymethylglutaryl-CoA synthase, cytoplasmic; MVK, Mevalonate kinase; NCSTN, Nicastrin; NSDHL, Sterol-4-alpha-carboxylate 3-dehydrogenase, decarboxylating; ATP6V0D1, V-type proton ATPase subunit d 1; DHCR7, 7-dehydrocholesterol reductase; PCYOX1, Prenylcysteine oxidase 1; SCARB2, Lysosome membrane protein 2; LSS, Lanosterol synthase; RDH11, Retinol dehydrogenase 11; CYB5B, Cytochrome b5 type B; FASN, Fatty acid synthase; SQSTM1, Sequestosome-1; and ACSL3, Fatty acid CoA ligase 3.

Assembly of viral replication complexes and MERS-CoV progeny virions requires large quantities of specific lipid species to support rearrangement of intracellular membranes and the manufacture of viral particles; hence, we hypothesized that some smaller component of the lipid-related cellular machinery must be upregulated during infection. Since the data across cell types and human donors had variable outcomes, we used a simple counting procedure to identify the strongest upregulated signals among lipid-related proteins (i.e., the pooled three gene sets mentioned above), with the goal of identifying consensus candidates most likely to be important for MERS-CoV replication. The number of experimental conditions (experimental condition = single time point, in single-cell type from a single donor, zero time points excluded, maximum = 36) in which each lipid-associated protein was upregulated following MERS-CoV infection was counted. ACSL3 had the most counts (12) with sequestosome 1 (SQSTM1) having the next highest counts (10) (Fig. 3A). ACSL3 mediates fatty acid oxidation, leading to the synthesis of TG and is localized to and mediates functions within lipid droplets (LD) and the endoplasmic reticulum (22). SQSTM1/p62 is the prototypical autophagy receptor and one of its functions is to selectively target ubiquitinated proteins for autophagy-mediated degradation (23). To determine if certain donor/cell types were dominating the counts, we collapsed the time points and counted again (Fig. 3B), allowing a count for a donor/cell-type combination if a protein was upregulated at least once across the time points (maximum = 9). From this approach, the highest count was again from ACSL3 (6), and SQSTM1 (4) was again second (tied with fatty acid synthase [FASN]). We next assigned counts when a lipid-associated protein was upregulated at any time post-MERS-CoV infection for each cell type (Fig. 3C, maximum = 3). ASCL3 and FASN were the only two lipid-related proteins with increased expression at least once in all three primary human lung cell types following MERS-CoV infection, with SQSTM1 being one of 16 proteins upregulated in two of the three cell types. Finally, we determined the summed significance (see methods for description) for each lipid-related protein across all conditions (Fig. 3D) and found SQSTM1 to have the highest signal, with ACSL3 close behind with the second highest score. For comparison to another pathogenic human coronavirus, we performed the same analysis as in Fig. 3D using data sets derived from severe acute respiratory syndrome coronavirus (SARS-CoV)-infected primary human lung cells (Fig. S4) and found that SQSTM1 again had the strongest upregulated signal, but ASCL3, although upregulated, was not among the proteins with the highest signal. SQSTM1 behavior (Fig. S5) also showed cell type-dependent responses: general downregulation in FB, an ambiguous response in HAE, and stark general upregulation in MVE following MERS-CoV infection.

**Fig 4:**
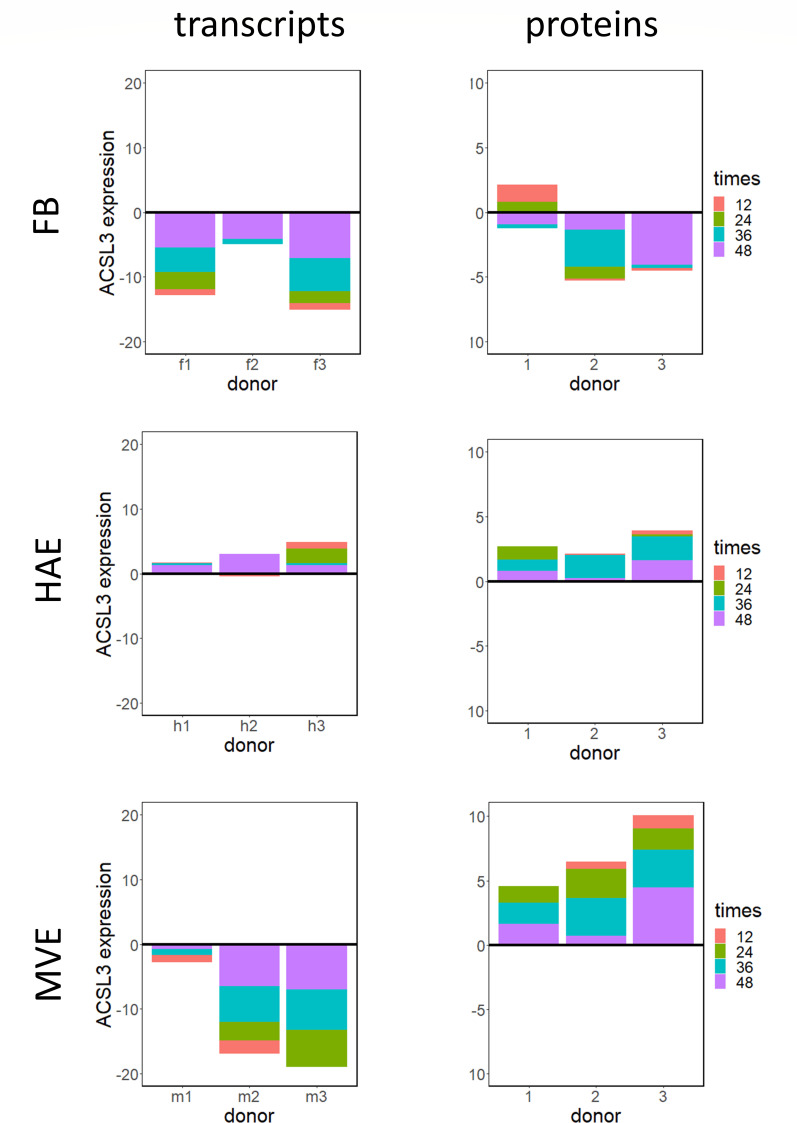
Differential expression of ACSL3 transcripts and proteins. ACSL3 transcript (left panels) and protein (right panels) levels were plotted according to the degree of differential expression at each time point (indicated with color) using the −log10 of the significance *P*-value, with upregulation placed above the line and downregulation placed below the line. Each individual-colored block in the stacked bar graph indicates the significance level for ACSL3 gene/protein for each individual time point (the colored blocks are stacked into a single bar, rather than being individual bars that each extend from/to the zero line). ACSL3, acyl-CoA-synthase long chain family member 3; FB, f-fibroblast; MVE, m-microvascular endothelial cells; and HAE, h-human airway epithelial cells cultures.

Next, we examined the overall expression patterns of ASCL3 transcripts and proteins across all experimental conditions (Fig. 4). Interestingly, the pattern was distinct across all three cell types. In MERS-CoV-infected FB, ACSL3 transcripts and proteins had decreased expression (although Ddonor 1 had increased levels of differentially expressed ACSL3 protein at early times post-infection). In contrast, ACSL3 transcripts and proteins were upregulated in infected HAE, whereas ACSL3 transcript expression was decreased and protein expression was increased when compared with mock-infected cells in MERS-CoV-infected MVE. Because of the role of ACSL3 in TG synthesis and its association with LD, we also examined the transcription levels for additional genes known to contribute to the synthesis of TG and cholesterol esters (Fig. S6), which are important components of LDs. The transcript expression levels of 10 of the genes encoding these enzymes, plus perilipin2 (PLIN2), an LD marker, are shown in Fig. S6. While transcripts for these genes were often increased, they were essentially all either undetectable or unchanged at the protein level (data not shown). Along with ACSL3, ACSL4, and ACSL5 proteins were detected in some samples and did have increased expression (Fig. S7); however, this effect was mostly limited to FB and MVE cells.

### ACSL3-mediated fatty acid esterification of long-chain fatty acids is important for MERS-CoV replication

**Fig 5:**
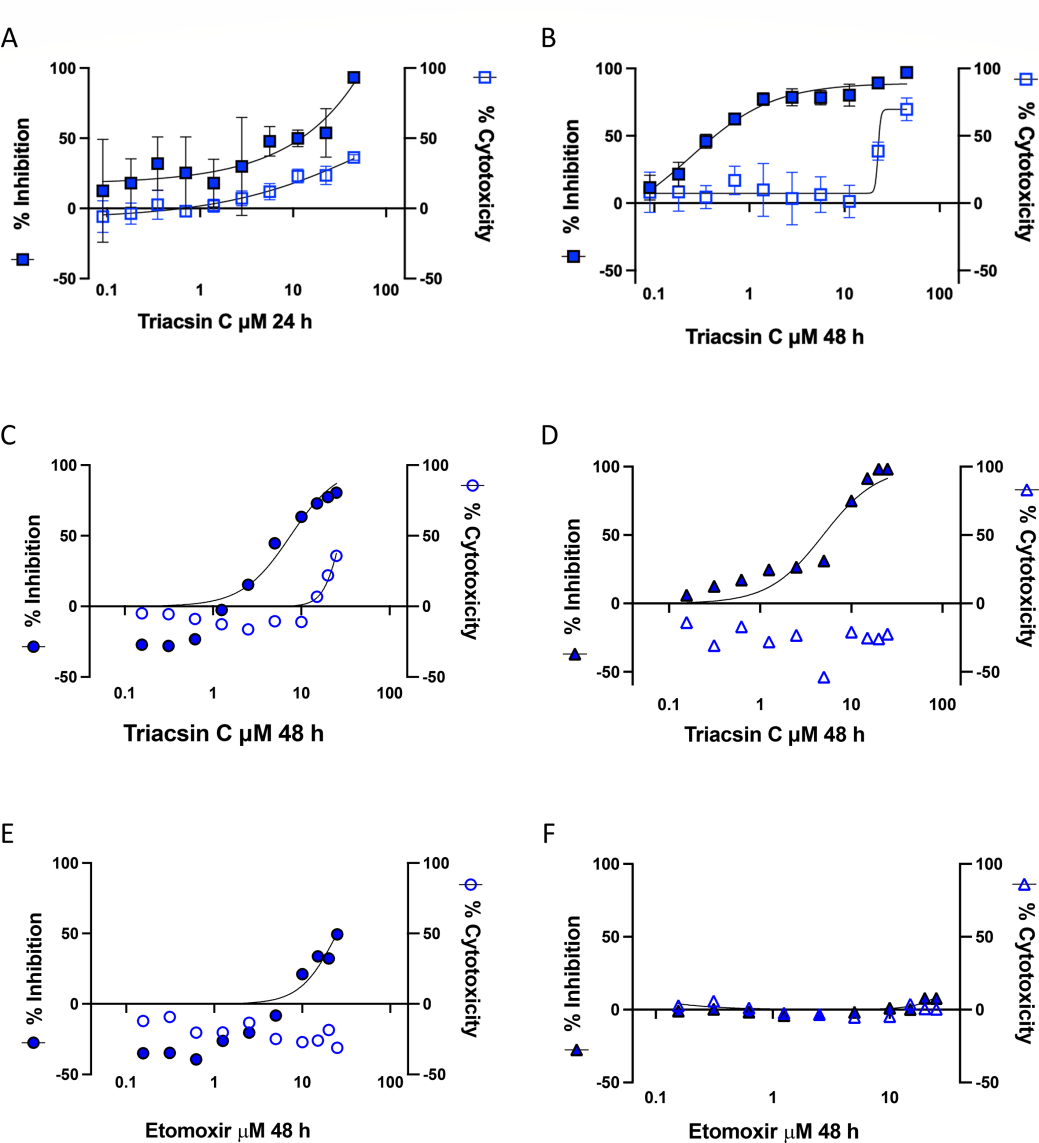
MERS-CoV replication requires ongoing acyl-CoA formation and fatty acid oxidation. Triacsin C (**A–D**) or etomoxir (**E-F**) was used to treat cells for 24 (**A**) to 48 (**B–F**) h during either MERS-LUC infection (**A–C, E**) or SARS2-LUC infection (**D, F**). A range of doses was used to test drug cytotoxicity (as assessed by ATP levels, unfilled symbols) and percent inhibition for MERS-LUC or SARS2-LUC expressing nanoluciferase (as assessed by luciferase production, filled symbols) as shown. (**A**) MERS-LUC-infected primary human lung fibroblasts (Since the regression curve did not manifest an inflection point, no EC50 could be calculated. The dose where the curve crosses 50% inhibition is ~14.09 μM, CC50 ~55.78 μM). (**B**) MERS-LUC-infected primary human lung microvascular endothelial cells (EC50 ~0.24 μM, CC50 ~22.50 μM). (**C**) MERS-LUC infected immortalized human lung epithelial cells (Calu3 EC50 ~3.50 μM, CC50 >25 μM). (**D**) SARS2-LUC infected immortalized non-human primate epithelial cells (Vero E6, EC50 ~7.86 μM, CC50 >25 μM). (**E**) MERS-LUC-infected immortalized human lung epithelial cells (Calu3, EC50 ~9.97 μM, CC50 >25 μM). (**F**) SARS2-LUC infected immortalized non-human primate epithelial cells (Vero E6, EC50 NA, CC50 >25 μM) EC50- half-maximal effective concentration; CC50- half-maximal cytotoxic concentration.

To determine if inhibition of fatty acid metabolism via ACSL3 alters levels of MERS-CoV replication, inhibitor studies were performed in mock-infected and MERS-CoV-infected human lung cells as previously described (9). Triacsin C is a potent inhibitor of long-chain fatty acid acyl-CoA synthetase enzymes, such as ACSL3, that mediate the transition of fatty acids into acyl-CoA; an early step in *de novo* TG synthesis (24) (Fig. 5A through D). Etomoxir inhibits the activity of carnitine palmitoyltransferase-1, a mitochondria-associated enzyme downstream of ACSL3 in the fatty acid beta-oxidation pathway (Fig. 5E and F)—a pathway distinct from TG synthesis and previously shown not to be involved in SARS-CoV-2 replication (25). Cytotoxicity was assessed in drug-treated, mock-infected cells by monitoring levels of ATP as an indicator of cell viability. As a surrogate for viral replication, levels of nanoluciferase expression were determined in cells treated with drug and infected with a recombinant strain of MERS-CoV-expressing nanoluciferase (MERS-LUC). Primary human lung FB and MVE and immortalized human lung epithelial cells (Calu3) were simultaneously infected with MERS-LUC and treated with a range of doses of Triacsin C, monitoring nanoluciferase expression as a surrogate for viral replication. Calu3 cells were used as a surrogate for primary human airway epithelial cells as they are permissive for MERS-CoV replication and can be differentiated into polarized bronchial epithelial cell cultures (26). To build upon the previous studies to determine the efficacy of etomoxir treatment for SARS-CoV-2 infection (25), immortalized human lung epithelial cells (Calu3) were infected with MERS-LUC, and in parallel, Vero E6 cells were infected with SARS2-LUC in the presence or absence of a range of doses of etomoxir. Etomoxir was not cytotoxic in the absence of infection at the dose ranges tested (Fig. 5E and F). Doses of Triacsin C >20 μM were cytotoxic for epithelial cells and MVE, but doses >1 μM were cytotoxic for FB (Fig. 5A through C). Triacsin C treatment reduced the levels of nanoluciferase expression in human lung cells in a dose-dependent pattern at subcytotoxic levels, suggesting that blocking ACSL3 activity does reduce MERS-CoV replication in all three cell types following MERS-LUC infection (Fig. 5A through C). Interestingly, etomoxir treatment also reduced the levels of nanoluciferase expression following MERS-LUC infection (Fig. 5E). Triacsin C treatment of SARS2-LUC-infected immortalized epithelial cells reduced luciferase expression, supporting the fact that the highest doses could restrict replication, but no effect of etomoxir treatment was observed, confirming previous findings (25, 27). These results support the importance of long-chain fatty acid acyl-CoA synthetase activity but not fatty acid beta-oxidation for SARS-CoV-2 replication and suggest that fatty acid beta-oxidation may be more important for MERS-CoV replication than for SARS-CoV 2 (Fig. 5E and F) (25).

### MERS-CoV infection significantly alters the expression of three predominant lipid subclasses in a cell type-dependent pattern

**Fig 6:**
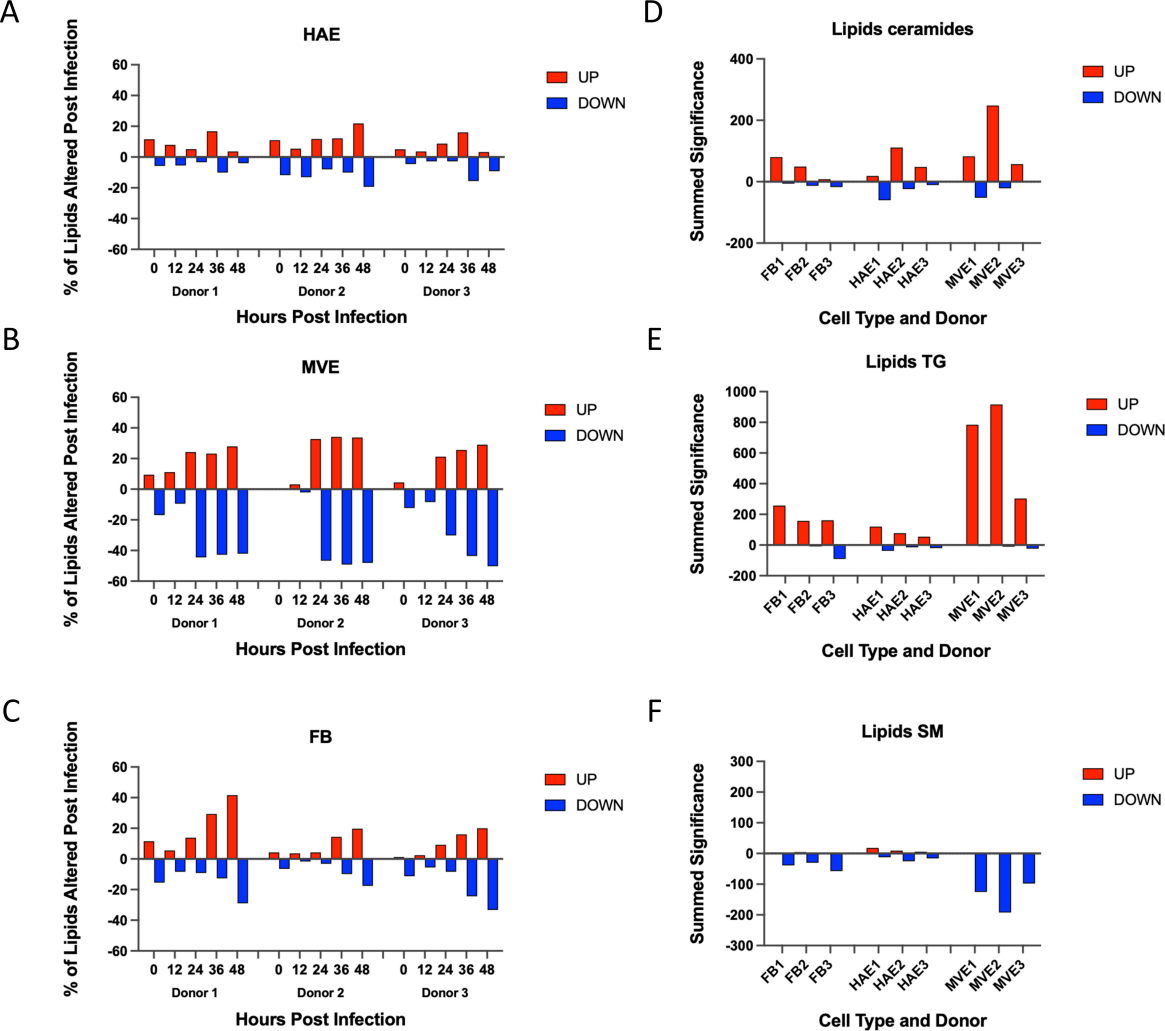
Primary human lung cell lipid species differential expression following MERS-CoV infection. (**A–C**) Percentage of detected lipid species that were up- (red) or down-regulated (blue) in human airway epithelium (**A,** HAE), microvascular endothelial cells (**B,** MVE), or fibroblasts (**C,** FB). Data are shown for all three human donors. (**D–F**) Summed significance of changes in specific lipid classes across donors and tissue types. Summed significance is the sum of −log10 *P*-values of changes of all species of a particular class across time points, represented for each tissue type and donor. Panels D–F show the results for ceramides (**D**), triacylglycerides (**E,** TG), and sphingomyelins (**F,** SM).

Previous studies have suggested that increased levels of the lipid ceramide enhanced SARS-CoV-2 replication and that patients who were taking functional inhibitors of acid sphingomyelinase (FIASMA) inhibitors had reduced disease outcomes (4, 28–31). Interestingly, MERS-CoV replication in primary human lung cells led to increased intracellular ceramide levels, especially in MVE (9). To expand our understanding of how MERS-CoV infection alters the levels of lipid species, the lipidomics data sets from FB, HAE, and MVE for each donor were condensed by time point, and differentially expressed lipid species were compared. Heatmaps of the expression levels of individual species of ceramide, TG, and sphingomyelins (SM) are shown in Fig. S8, building upon our previous results. Lipids with increased or decreased expression following MERS-CoV infection were detected in all donors for all three cell types and at each time point post-infection, with considerable variation displayed across cell types. Overall trends in lipid species found to be differentially expressed were similar between the donors (Fig. 6A through C), while differential abundance results from a representative time point (36 h) for each of the cell types (all three donors combined, Fig. S9) supported the analysis in Fig. 6. In our previous study, we determined the enrichment of lipid species within lipid subclasses for each condition (9) and plotted species-level changes in ceramide and TG levels following MERS-CoV infection across the infection time course for all three donors. To identify the most consistent expression trends of lipid species between donors, we calculated the cumulative significance of all increases and decreases for three classes of lipids (see Materials and Methods, Fig. 6D through F) and plotted them by cell type and donor. Our new analysis highlighted that in contrast to ceramide and TG, SM species had lower levels following MERS-CoV infection for all three cell types (Fig. 6F). As SM can be converted into ceramide, reduced levels of SM and increased levels of ceramide were consistent with a sphingomyelinase (SMase)-dependent pathway(s) for ceramide synthesis during MERS-CoV infection. However, it is unknown whether ceramide synthesis is critical for MERS-CoV replication, although it has been shown to be required for SARS-CoV-2 replication (4).

### Ceramide synthesis inhibitor studies

**Fig 7:**
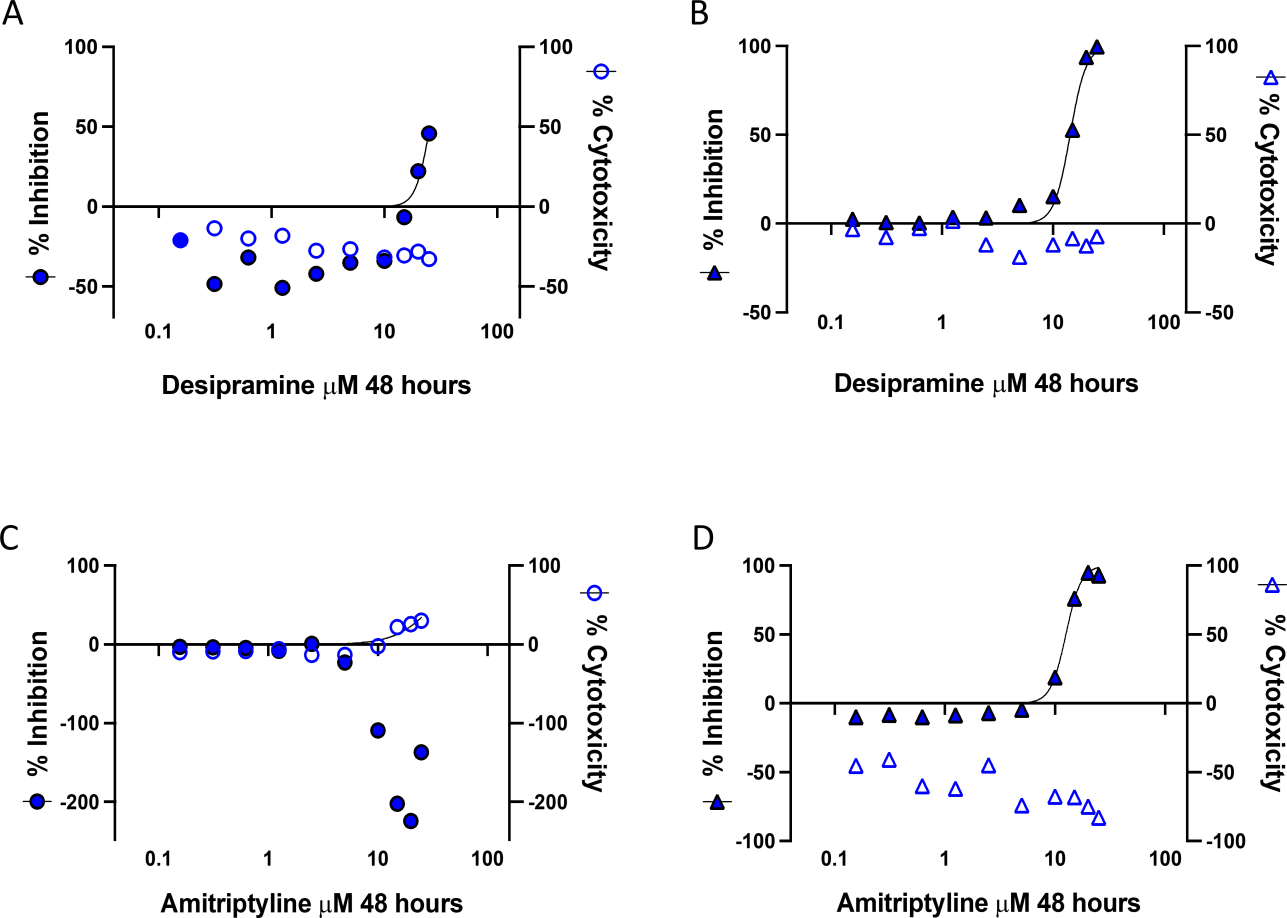
Ceramide synthesis is required for MERS-CoV replication. To determine the importance of sphingomyelinase-dependent ceramide synthesis for CoV replication, we evaluated the efficacy for several FIASMA drugs in MERS-CoV or SARS-CoV-2-infected epithelial cells. Desipramine (**A-B**) and amitriptyline (**C-D**) were used to treat lung cells for 48 h during either MERS-CoV infection (**A, C**) or SARS-CoV-2 infection (**B, D**). A range of doses was used to test drug toxicity (as assessed by ATP levels, unfilled symbols) and virus replication (as assessed by nanoluciferase production, filled symbols) as shown. (**A**) MERS-LUC infected immortalized human lung epithelial cells (Calu3, EC50 ~18.66 μM, CC50 >25 μM). (**B**) SARS2-LUC-infected immortalized non-human primate epithelial cells (Vero E6, EC50 ~15.04 μM, CC50 >25 μM). (**C**) MERS-LUC-infected immortalized human lung epithelial cells (Calu3, EC50 NA, CC50 >25 μM). (**D**) SARS2-LUC-infected immortalized non-human primate epithelial cells (Vero E6, EC50 ~11.83 μM, CC50 >25 μM). EC50, half-maximal effective concentration; and CC50, half-maximal cytotoxic concentration.

**Fig 8:**
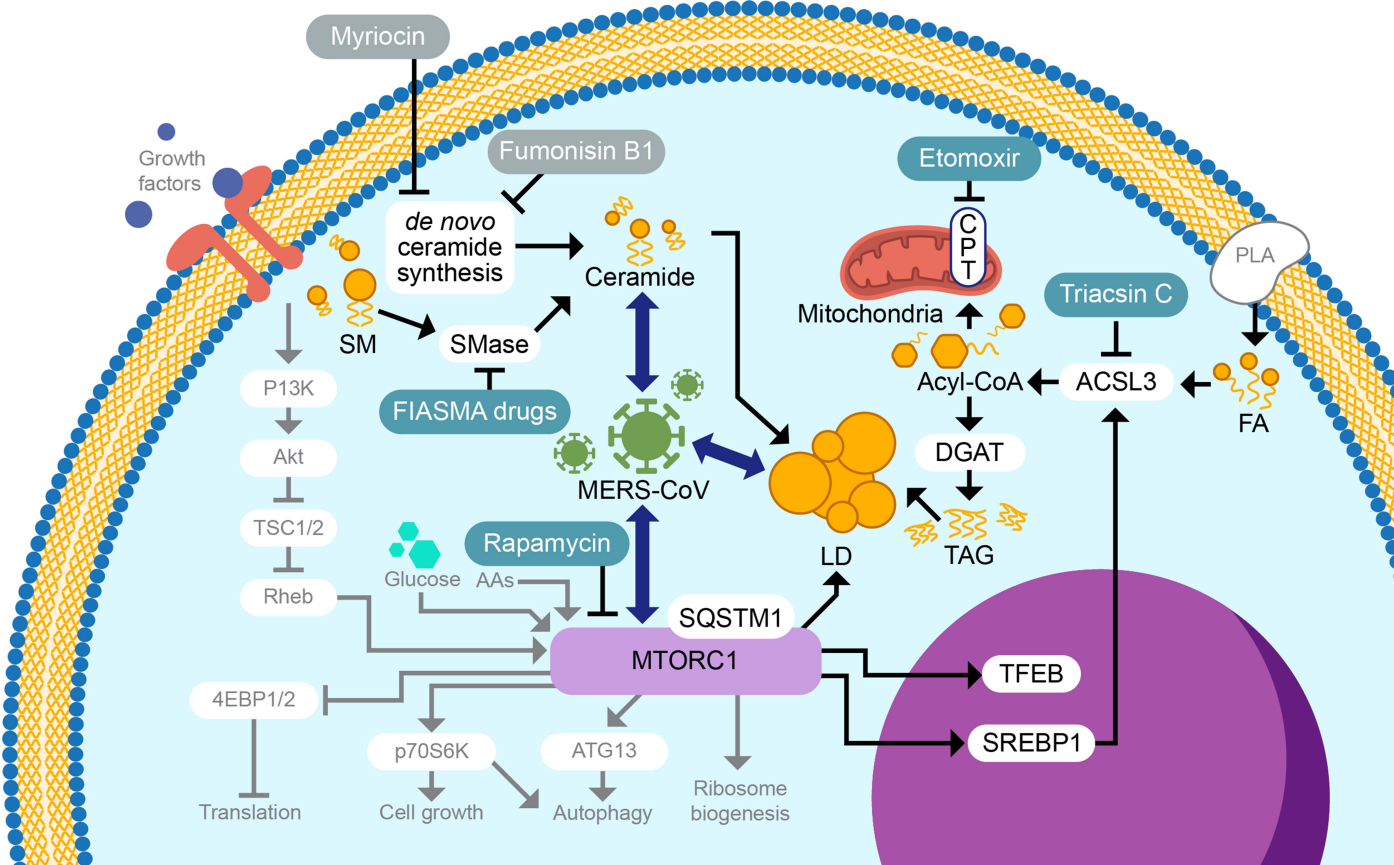
Model of lipid-related cell signaling during MERS-CoV infection. Under normal conditions, growth factors, amino acids, glucose, and other factors stimulate the mTORC1 complex through PI3K and Akt. mTORC1, which is inhibited by rapamycin, then stimulates a general growth state through the activation of translation, transcription, and lipid production. This signaling can eventually lead indirectly to autophagy. The results from rapamycin treatment support the possibility that MERS-CoV replication influences the mTORC1 complex to produce a subset of lipids, possibly through TFEB and SREBP1; however, additional studies will be required to conclusively show this. Subsequent ACSL3 upregulation catalyzes the conversion of fatty acids to acyl-CoA, which can be diverted to mitochondria for energy utilization through beta-oxidation (facilitated by carnitine palmitoyl transferase [CPT] and inhibited by etomoxir). Through DGAT, acyl-CoA is also utilized for TAG production and may then be incorporated into LDs. LDs represent a requirement for viral propagation, as does ceramide, which, our studies suggest, is produced through SMase, as opposed to *de novo* ceramide synthesis. PI3K, phosphoinositide 3-kinase; Akt, Ak strain transforming; TSC 1 /2, tuberous sclerosis; Rheb, Ras homolog enriched in brain; 4EBP 1 /2, eukaryotic translation initiation factor 4E 1/2; p70S6K, ribosomal protein S6 kinase beta 1; ATG13, autophagy-related gene 13; mTORC1, mammalian target of rapamycin complex 1; SQSTM1, sequestosome 1; AAs, amino acids; FIASMA, functional inhibitors of acid sphingomyelinase; SM, sphingomyelin; SMase, sphingomyelinase; MERS-CoV, Middle East respiratory syndrome coronavirus; TFEB, transcription factor EB; SREBP1, sterol regulatory element binding protein 1; LD, lipid droplet; TAG, triacylglyceride; DGAT, diacylglycerol O-acyltransferase; CPT, carnitine palmitoyltransferase; ACSL3, acyl-CoA synthetase long chain family member 3; FA, fatty acids; and PLA, phospholipase A.

Previous studies in Zika virus-infected cells demonstrated a dependence on ceramide production through *de novo* synthesis (32). To investigate the role of *de novo* ceramide production during MERS-CoV replication, inhibitor studies were performed in mock-infected and MERS-CoV-infected primary human lung cells. Intracellular ceramide production occurs by two main mechanisms (33): (i) *de novo* synthesis and (ii) via SMase breaking down SM into ceramide (Fig. 8). *De novo* synthesis can be inhibited by the thermophilic fungal inhibitor Myriocin, which inhibits serine palmitoyl-CoA transferase at the first step of *de novo* ceramide synthesis or by the mycotoxin Fumonisin B_1_ at the last step where ceramide synthase mediates the conversion of sphingosine to ceramide. Treatment of MERS-CoV-LUC-infected cells with a range of doses of Myriocin or Fumonisin B1 resulted in no change to cellular viability or levels of nanoluciferase expression (Table 1; Fig. S10), suggesting that *de novo* synthesis is not a critical pathway of ceramide production during MERS-CoV replication. We next investigated whether the increase in ceramide production and concomitant reduction in SM species results from SMase activity. FIASMA is a large group of pharmaceutical compounds that inhibit acid SMase (ASMase) activity and potentially play a role in reducing COVID-19 disease severity (28, 34). Treatment with the FIASMA inhibitor desipramine has been demonstrated to reduce the levels of ceramide production *in vivo* (35, 36). The treatment outcomes with FIASMA inhibitors have been well established for SARS-CoV 2 (29), but they are yet to be tested in MERS-CoV infection. ASMase-targeted inhibitor studies were performed as described above in immortalized human lung cells. We tested four different FIASMA drugs and found that one of these, desipramine, reduced detectable levels of nanoluciferase expression at the two highest doses with no detectable cytotoxicity in doses ≤25 μM (Table 1; Fig. 7A and B), suggesting that ASMase activity is important for MERS-CoV replication. Treatment of MERS-LUC-infected immortalized human lung epithelial cells with amitriptyline, imipramine, or sertraline did not reduce the levels of nanoluciferase expression at any dose tested (Fig. 7C; Table 1). Detectable levels of nanoluciferase expression were reduced following desipramine and amitriptyline treatment of immortalized non-human primate epithelial cells infected with SARS2-LUC (Fig. 7B and D), supporting the importance of ASMase activity for CoV replication. These results confirmed similar studies to determine the efficacy of FIASMA inhibitors to reduce SARS-CoV-2 replication; however, not all FIASMA compounds tested in our assay reduced nanoluciferase expression (Table 1) (29, 30).

**TABLE 1:** Summary of evaluated ceramide synthesis inhibitors^*a*^

Inhibitor	Type of ceramide production	Reduced MERS-CoV replication	Reduced SARS-CoV-2 replication	Cytotoxic
Myriocin	*De novo*	No	Not tested	No
Fumonisin B_1_	*De novo*	No	Not tested	No
Amitriptyline	FIASMA	No	Yes	≥20 μM
Desipramine	FIASMA	Yes	Yes	No
Imipramine	FIASMA	No	Not tested	≥ 50 μM
Sertraline	FIASMA	No	Not tested	≥ 10 μM

^*a*^ The table summarizes (i) the list of inhibitors tested, (ii) whether the inhibitor prevents *de novo* or acid sphingomyelinase-mediated synthesis, (iii) if the inhibitor prevented MERS-CoV or SARS-CoV-2 replication, and (iv) whether the inhibitor was cytotoxic in the cells tested. If the compound was cytotoxic, the concentration at which cytotoxicity occurred is noted. See Materials and Methods for the cell types tested for each inhibitor.

### Probing the potential involvement of mTORC1 in distinct cell types

Transcriptomic and proteomic analyses of gene/protein sets with lipid synthesis-associated functions revealed decreased expression following MERS-CoV infection (Fig. 2; Fig. S1 and S2). The mTORC1 protein complex is a major point of regulation of many cellular processes, including transcript, protein, and lipid synthesis, as well as growth, motility, survival, and autophagy (37, 38). Virus-host interactions are complex and stem from combinations of viral and host-driven mechanisms, a subset of which could be mediated by the mTORC1 complex, as this complex functions as a control point for major disruptions of metabolic processes, such as viral infections (19, 39–44), and previous work demonstrated involvement of PI3K/mTORC1-related kinase signaling in SARS-CoV 2 and MERS-CoV (45). Cellular pathways regulated by mTORC1 function are related to several proteins of interest in our analysis, including TFEB, SQSTM1, and SREBP (Fig. 3 and 8; Fig. S1, S2, and S5). One possible scenario is that MERS-CoV infection favors the preservation of mTORC1 signaling to maintain sufficient protein and lipid synthesis for viral replication. Therefore, inhibition of mTORC1 would be expected to have a negative effect on MERS-CoV replication. In previous work, MERS-CoV and SARS-CoV-2 replication were shown to be blocked by the mTORC1 inhibitor rapamycin (39, 41, 43). However, the earlier MERS-CoV study was performed in Huh7 cells, which are derived from a hepatic tumor (41) and are not as biologically relevant for MERS-CoV infection as primary human lung cells. We therefore tested the effects of rapamycin on MERS-CoV replication in primary human lung FB and MVE cells (Fig. S11), as well as in human lung epithelial cells. No cytotoxicity from rapamycin treatment was detected at any dose administered in all three cell types (data not shown). A high dose of rapamycin reduced MERS-LUC nanoluciferase expression at 24 and 48 h post-infection/treatment in FB (Fig. S11A). No significant reduction in nanoluciferase expression was observed in MVE cells (Fig. S11B). In human epithelial cells, a dose of 30 μM rapamycin was required to reduce the levels of MERS-LUC nanoluciferase expression (Fig. S11C). Although not conclusive, these results suggested that host cells may attempt to downregulate metabolic resources; successful MERS-CoV replication may require the preservation of some portion of mTORC1 signaling, although the need for mTORC1 appeared to vary between cell types. Further work will be needed to test this hypothesis.

## DISCUSSION

Using a combined approach of integrated functional enrichment with targeted pharmacological infection phenotype perturbation, we identified and successfully tested candidate key lipid regulatory pathways that likely represent critical therapeutic targets for MERS-CoV infections. Focusing on transcripts, lipids, and proteins with lipid-associated functions expanded our previous analysis and identified additional cellular pathways that are essential for CoV replication. MERS-CoV infection resulted in decreased expression of transcripts linked to functions within the lysosome, SREBP, and TFEB cellular GO pathways. Lysosomes contain a variety of hydrolases that mediate lipid catabolism, facilitate endocytosis and the generation of autophagosomes, and regulate autophagy (46, 47). Dysregulation of lysosome-autophagosome fusion has been proposed as a way SARS-CoV-2 avoids degradation during infection (48). SREBP-responsive genes are a family of transcription factors that regulate the expression of enzymes required for the synthesis of endogenous cholesterol, fatty acids, triacylglycerol, and phospholipids to modulate the levels of intracellular lipid species and are associated with LD accumulation (49, 50). Previous studies identified SREBP as a key host factor for MERS-CoV replication (51). SARS-CoV-2 infection has been demonstrated to activate SREBP, leading to increased levels of LDs (50). TFEB is a transcription factor that regulates the expression of genes required to facilitate lysosomal biogenesis and autophagy, regulate lipid catabolism, and degrade LDs (46, 52). TFEB activation was recently linked with beta-coronavirus replication (53). Our current study provided clues as to how MERS-CoV replication may control intracellular LD formation, autophagy pathways, and regulation of lipid synthesis pathways to mediate virion production, release, and transmission.

### Lipids

Further in-depth analyses of the lipidomics data set revealed that SM species abundances (Fig. 6F; Fig. S7C) were strikingly decreased. Our data support that SM is a likely precursor for increased ceramide detected during MERS-CoV replication, which would require active ASMase. ASMase functions have been linked to a variety of other virus strains, including Ebola virus, measles virus, and rhinovirus, predominantly for enhancing virus entry (54–56). While it is unclear why only one FIASMA drug was effective (Table 1), this result is not completely surprising, since these drugs are structurally diverse and have shown varying efficacies in other test conditions, including modulating COVID-19 severity (57). Future studies investigating the inhibition of other SMases, such as neutral SMases, could also be useful. Larger quantities of ceramide may allow for ceramide accumulation in the ER, contributing to the buildup of misfolded proteins and leading to the activation of the UPR (58, 59). The presence of ceramide in ER membranes may also stimulate the formation of LD (60), supporting a linkage between increased intracellular ceramide levels and LD formation for optimal MERS-CoV infection (Fig. 8). This suggests that the enhanced levels of ceramide through ASMase-dependent ceramide synthesis or other mechanisms and/or the apoptotic state may contribute to the ideal intracellular environment for MERS-CoV replication.

### ACSL3

In a background of predominantly downregulated protein expression, our analysis identified ACSL3 and SQSTM1 as the most significantly upregulated proteins with lipid-related functions in MERS-CoV-infected primary human lung cells. In combination with the dramatic increase in TG species in multiple cell types, this suggests that ACSL3 activity is likely key for successful MERS-CoV replication. SARS-CoV-2, Ebola virus, and picornavirus infections also lead to alterations in TG abundance in models of infection (4, 61, 62) and in human plasma from Ebola virus-infected individuals (62). Dengue and polio virus-infected cells also have increased the expression of ACSL, and in COVID-19 patients, high levels of ACSL3 can be indicative of enhanced disease severity (27, 63, 64). During lipid biosynthesis, ACSLs convert free fatty acids into fatty acyl-CoA, which is one of the first steps in TG synthesis (65), suggesting a mechanism for general TG upregulation following MERS-CoV infection.

Together with cholesterol esters, TGs are one of the major components of LDs, which are energy reservoirs for cells and important for viral replication as previously demonstrated for SARS-CoV-2 (66). LDs were previously thought to be simple lipid storage units but are now known to play key roles in a variety of cellular response pathways, including the immune response, stress response, and protein folding and storage (67, 68). While we were unable to consistently detect PLIN2 (a protein marker for LDs) in our proteomics, ACSL3 has been found to be associated with LDs (22), which are prominently seen in MERS-CoV-infected cells (51, 69). Thus, ACSL3 may represent a key component of the host machinery perturbed by MERS-CoV infection, as it manipulates the intracellular environment to be favorable for virus production. LD numbers increase during most, but not all, viral infections, including MERS-CoV and SARS-CoV-2, hepatitis C virus, influenza virus, zika virus, herpes simplex virus 1, and dengue virus (66, 67, 70–72). Interestingly, SARS-CoV-2 virions have been demonstrated to be physically associated with LD, and inhibition of LD formation reduces SARS-CoV-2 replication (66). Since ACSL3 resides within LD, is required for LD nucleation, and facilitates lipid storage within LDs (22), we reasoned that inhibition of ACSL3 using Triascin C could reduce MERS-CoV replication most likely by decreasing TG levels and LD formation, representing a possible therapeutic avenue (Fig. 5 and 8). Interestingly, Triacsin C activity was identified by Santos-Beneit et al. (73) as a promising antiviral drug to reduce COVID-19 severity (73). Treatment with Triacsin C significantly impacted MERS-CoV infection, supporting our hypothesis. Future studies will focus on the mechanisms that upregulate ACSL3 and TG expression during MERS-CoV infection.

Of note, ACSL3 is also the acyl-CoA synthetase that has increased expression in several types of lung cancer (71, 74), suggesting that this enzyme may play a key role in enhancing cell viability following major stresses such as infection or cancerous transformation. In cancerous cells, especially aggressive cancer types, increased demands for cellular energy sources lead to increased functionality and overall numbers of lysosomes and LD (75–77), positioning the cell to continue proliferating under stressful conditions. As MERS-CoV-infected cells also have increased numbers of LD and enhanced TG synthesis (78), these findings support a link between the intracellular environments found in lung cancer and MERS-CoV infection and suggest interesting mechanistic parallels between them. Future studies will be required to explore this link more thoroughly.

ACSL3 resides not only in LD but also inside the endoplasmic reticulum (22), both key intracellular locations for different stages of MERS-CoV replication (9, 51, 79). Previous studies demonstrated that activation of the UPR (which occurs in the ER) is required for MERS-CoV replication, supporting the notion that protein synthesis and proper protein folding are required for progeny virion release (9). As the endoplasmic reticulum is also the site for lipid and carbohydrate synthesis, as well as LD biogenesis, we predict that MERS-CoV replication alters the functions of additional endoplasmic reticulum-based cellular pathways.

SQSTM1/p62 is a ubiquitin-binding protein that is degraded along with targeted proteins during normal autophagy, and one of its functions is to selectively target ubiquitinated proteins for autophagy (23, 80). In this way, its cellular abundance is negatively correlated with activation of autophagy and is considered to be a prototypical autophagy marker. SARS-CoV-2 proteins, particularly the membrane protein (M), nonstructural protein 5 (NSP5), and open reading frame protein 3a (ORF3a), have been shown to interact with SQSTM1/p62 (48, 80, 81), supporting the importance of this protein in coronavirus replication. The increased expression of SQSTM1 in MERS-CoV-infected MVE supports previously published studies (44, 82) that demonstrated MERS-CoV M and ORF4b proteins play roles in enhanced SQSTM1 differential expression. The SQSTM1 levels we observed suggested that autophagy was appropriately activated in FB (80) and was being dysregulated in MVE following MERS-CoV infection (80) and may provide clues as to why infection ultimately results in apoptotic cell death in MVE, but not in FB (9). The interplay between coronaviruses and autophagy is somewhat controversial, and the exact interactions between coronavirus proteins and host proteins still need to be defined. Formation of coronavirus-induced double membrane vesicles, required for replication complex formation, is independent of active autophagy; however, numerous publications support the hypothesis that coronaviruses interact with autophagy-associated proteins during replication and appear to induce both virophagy (an antiviral process that involves breaking down viral proteins) and mitophagy/lipophagy (processes that breakdown mitochondria and lipids, respectively) (48, 80, 81, 83). The distinct SQSTM1 responses of the three MERS-CoV-infected primary human lung cell types we tested suggest that autophagy, during coronavirus infection, is highly context-dependent.

Like ACSL3, mTORC1 has been shown to cause the formation of LDs, but in other cases, to cause their breakdown (84, 85). While our preliminary results using an mTORC1 inhibitor suggest its activity may be required for MERS-CoV infection, the complex role of mTORC1 in the regulation of lipid metabolism makes it difficult to determine whether its role is lipid-related. Despite this, the central role of mTORC1 in metabolism regulation makes it a useful target for future study, since many or even most viral infections are likely to either affect it or be affected by it, or both.

Our work highlights the importance of querying lipid species perturbations in a *side-by-side* manner with transcripts, proteins, and other omics platforms. Previously, we identified lipid changes that pointed toward an apoptotic response in infected cells, which was verified and further characterized by comparison with proteomics and transcriptomics, along with biochemical assays tracking markers of apoptosis and pharmacological inhibition studies (9). In the current study, we used trends in lipid changes to identify lipid synthesis pathways, alongside protein and transcript changes in lipid metabolic functions to identify key pathways necessary for MERS-CoV infection. Figure 8 illustrates the various lipid-associated pathways and mechanisms that we investigated as having a role in MERS-CoV replication based on our systems biology analysis of infection phenotypes. Our data support the importance of ASMase activity and demonstrate that activation of ACSL3-mediated fatty acid synthesis was required for effective MERS-CoV infection, likely via TG production and LD formation. Thus, synthesis and intracellular storage pathways of SM, ceramide, and TG represent key control points for MERS-CoV replication that should be explored in future studies. Our analysis suggested a model wherein coronaviruses perturb overall cellular metabolism to shift resources to the production of ceramides and triacylglycerides and support a strategy for targeting CoV replication through the inhibition of specific subsets of lipid metabolism.

## MATERIALS AND METHODS

### Primary human lung cells and immortalized cells

Primary human lung cells were isolated from the human conducting airway and processed as previously described (8). The lung cells were obtained under protocol 03-1396, approved by the University of North Carolina at Chapel Hill Institutional Review Board. Human lung microvascular endothelial cells were cultured in EGM-2 Endothelial Cell Growth Medium-2 (BulletKit, Lonza). Fibroblasts were cultured in Dulbecco's modified Eagle medium-H (DMEM-H) basal medium (Gibco), 1× penicillin-streptomycin (Gibco), and 10% fetal bovine serum (Cytiva-Hyclone). Vero 81 and E6 cells (kindly provided by R. Baric) were cultured in Dulbecco’s modified Eagle medium (DMEM Gibco) with 10% fetal bovine serum (Cytivia) and 1× antibiotic/antimycotic (Gibco). Calu 3 cells were cultured in Dulbecco’s modified Eagle medium (DMEM Gibco) with 20% fetal bovine serum (Cytivia) and 1× antibiotic/antimycotic (Gibco). For all cell types, fetal bovine serum concentrations were reduced to 4% for infection.

### Viruses and viral titration

MERS-CoV (MERS-LUC) and SARS-CoV-2 (SARS2-LUC)-expressing nano-luciferase were rescued from molecular clones, and stocks were kindly provided by R. Baric (8, 86, 87). Vero cells were used to generate MERS-LUC (Vero 81) and SARS2-LUC (Vero E6) viral stocks and quantitate viral titers for determining the MOI (8, 86, 87).

### Functional enrichment

Functional enrichment of transcriptomic, proteomic, and lipidomic samples first published in (9) was performed as previously described (9). To make the results visually manageable, all pathway enrichment results from transcriptomics with a −log10 *P*-value above 6 were retained. After arranging the output using hierarchical clustering, the results were divided into groups and sub-groups.

The kappa statistic for set overlap was used to cluster gene sets using set members appearing in the data (88). Two gene sets with a kappa overlap score of 0.35 or higher result in the sets being combined into one. When two or more sets were collapsed into one in this way, a name for the new set was chosen by first eliminating set names with a lower level in the GO hierarchy and then by choosing the most concise name (i.e., smallest number of characters). Summed significance was determined by summing the −log10 *P*-value of the significance of the difference between the infected one and control for all proteins/genes in a particular category (e.g., lipid-related). Negatively regulated features were then given a negative sign, so that up- and down-regulated items could be separately indicated.

### Gene sets

We used supplementary table 1 from Sardiello et al.’s publication as it contained a list of all known TFEB-responsive genes (89). We used table 1 from Palmieri et al.’s publication as it contained a list of all known lysosomal genes Table 1 (90). We used Tables 1 to 4 from Horton et al.’s publication for all known SREBP genes. Their Affymetrix probe IDs Tables 1 to 4 (91) were extracted and converted to symbols using https://davidbioinformatics.nih.gov/, and mouse symbols were converted to human symbols using https://www.informatics.jax.org/downloads/reports/HOM_MouseHumanSequence.rpt. The resulting three sets of genes can be found in File S3.

### Inhibitor studies

Recombinant MERS-CoV expressing nano-luciferase-infected Calu3 cells were evaluated at 24 and 48 h post-infection following treatment with a dilution series of amitriptyline (Sigma-Aldrich, 25–0.156 μM), desipramine (Sigma-Aldrich, 25–0.156 μM), imipramine (Sigma-Aldrich, 50–0.08 μM), sertraline (Sigma-Aldrich, 50–0.08 μM), triacsin C (Sigma-Aldrich, 25–0.156 μM), etomoxir (Sigma-Aldrich, 25–0.156 μM), rapamycin (Sigma-Aldrich, 30 μM), or remdesivir (TargetMol, 10–0.0195 μM) and were evaluated for cytotoxicity with drug treatment alone (cell viability assay, no virus developed using Promega CellTiterGlo) and viral replication (detection of nanoluciferase as a surrogate for replication developed using Promega NanoGlo). Recombinant MERS-LUC-infected MVE and FB cells were evaluated at 24 and 48 h post-infection following treatment with a dilution series of myriocin (Cayman Chemicals, 30–0.002 μM), fumonisin B1 (Sigma-Aldrich, 100–0.005 μM), triacsin C (Sigma-Aldrich, 44.4–1.16 μM), or rapamycin (Sigma-Aldrich, 10 μM) as described above. Recombinant SARS2-LUC infected Vero E6 cells were evaluated at 24 and 48 h post-infection following treatment with a dilution series of amitriptyline, desipramine, triacsin C, etomoxir, or remdesivir, with dosages and evaluation as described above. For all drug studies, diluted inhibitors and viral inoculum were added simultaneously at the beginning of the experiment. Viral inoculum was removed after 1 h, the cells were washed, and fresh media and drug dilutions were added and incubated for the indicated times post infection/treatment. All assay results were read by SpectraMax (Molecular Devices). The results are graphed as percent inhibition and toxicity (effective and cytotoxic concentration 50) as determined using GraphPad Prism.

Inhibition significance was performed using a one-sample *t*-test, testing if the collective percent inhibition replicates were significantly more than zero.

## Data Availability

All data sets used for these analyses can be found at data.pnnl.gov under the following numbers or using the indicated doi links: MERS-CoV infected fibroblasts-13095 (FB donor 1 https://doi.org/10.25584/LHVMFB001/1661935), 13096 (FB donor 2 https://doi.org/10.25584/LHVMFB002/1661936), 13097 (FB donor 3 https://doi.org/10.25584/LHVMFB003/1661937), MERS-CoV infected microvascular endothelial cells 13102 (MVE donor 1 https://doi.org/10.25584/LHVMMVE001/1661942), 13103 (MVE donor 2 https://doi.org/10.25584/LHVMMVE002/1661943), and 13104 (MVE donor 3 https://doi.org/10.25584/LHVMMVE003/1661944), MERS-CoV infected human airway epithelial cell cultures (HAE) 1661938 (HAE donor 1 https://doi.org/10.25584/LHVMHAE001/1661938), 1661939 (HAE donor 2 https://doi.org/10.25584/LHVMHAE002/1661939), 1661940 (HAE donor 3 https://doi.org/10.25584/LHVMHAE003/1661940).

## SUPPLEMENTAL MATERIAL

Supplemental Figures msphere.00523-25-s0001.pdf Figures S1 to S11.

File S1 msphere.00523-25-s0002.xlsx Table of names of all individual pathways, clusters and super-clusters in Fig. 2.

File S2 msphere.00523-25-s0003.xlsx Table of numerical −log10 *P*-values represented in Fig. S1 and S2.

File S3 msphere.00523-25-s0004.xlsx Table listing the members of each of the three gene sets used in Fig. S1 and S2.

ASM does not own the copyrights to Supplemental Material that may be linked to, or accessed through, an article. The authors have granted ASM a non-exclusive, world-wide license to publish the Supplemental Material files. Please contact the corresponding author directly for reuse.
