# Supplementary material for: Increased triacylglyceride and ceramide levels are key for MERS-CoV replication
Source: mSphere. 2026 Jan 15;11(2):e00523-25. doi: 10.1128/msphere.00523-25 (PMC12931271; doi:10.1128/msphere.00523-25)
Supplement: Supplemental Figures — Figures S1 to S11. [file msphere.00523-25-s0001.pdf]

Supplemental Figure 1

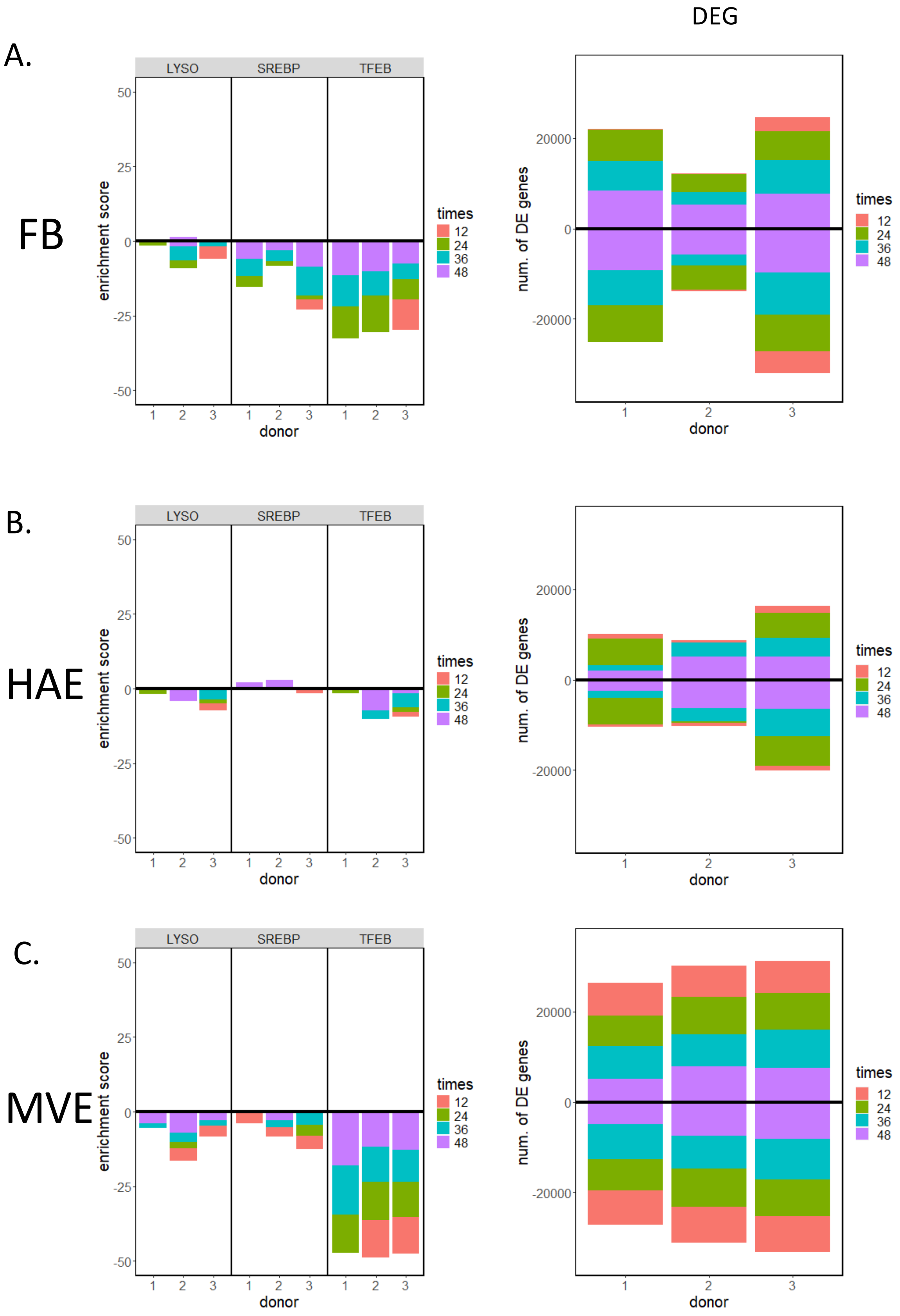

Supplement Figure 1. Gene expression enrichment in three distinct lipid-related gene sets. Enrichment of three distinct gene sets related to lipid-associated activity (lysosome proteins, SREBP-responsive genes, and TFEB-responsive genes) was determined for differentially expressed (increased or decreased expression) genes for each infection condition were identified. The results were plotted according to the degree of significance at each time point (indicated with color) and donor (indicated with x-axis numbers) using the -log10 of the significance p-value, with enrichment among up-regulated genes placed above the line and enrichment among down-regulated genes placed below the line, for each of the three cell types as indicated (left panels). Each individual-colored block in the stacked bar graph indicates the degree of enrichment for significantly changed genes for each individual time point (the colored blocks are stacked into a single bar). The result is that the total height of each multi-colored bar indicates the cumulative total degree of gene set enrichment across all time points for individual donors. To compare the downward trend of lipid-related processes to overall gene expression trends, the number of overall significantly up- and down-regulated genes was plotted at each time point (indicated with color) and donor (indicated with x-axis numbers) for each of the three cell types as indicated (right panels) with stacked blocks for each time point as in the left panels. LYSO-lysosomal; TFEB- transcription factor EB; SREBP1- sterol regulatory element binding protein 1; num- number; DE- differentially expressed; FB-fibroblast, MVE-microvascular endothelial cells, HAE-human airway epithelial cells cultures

Supplemental Figure 2

A.

FB

B.

HAE

C.

MVE

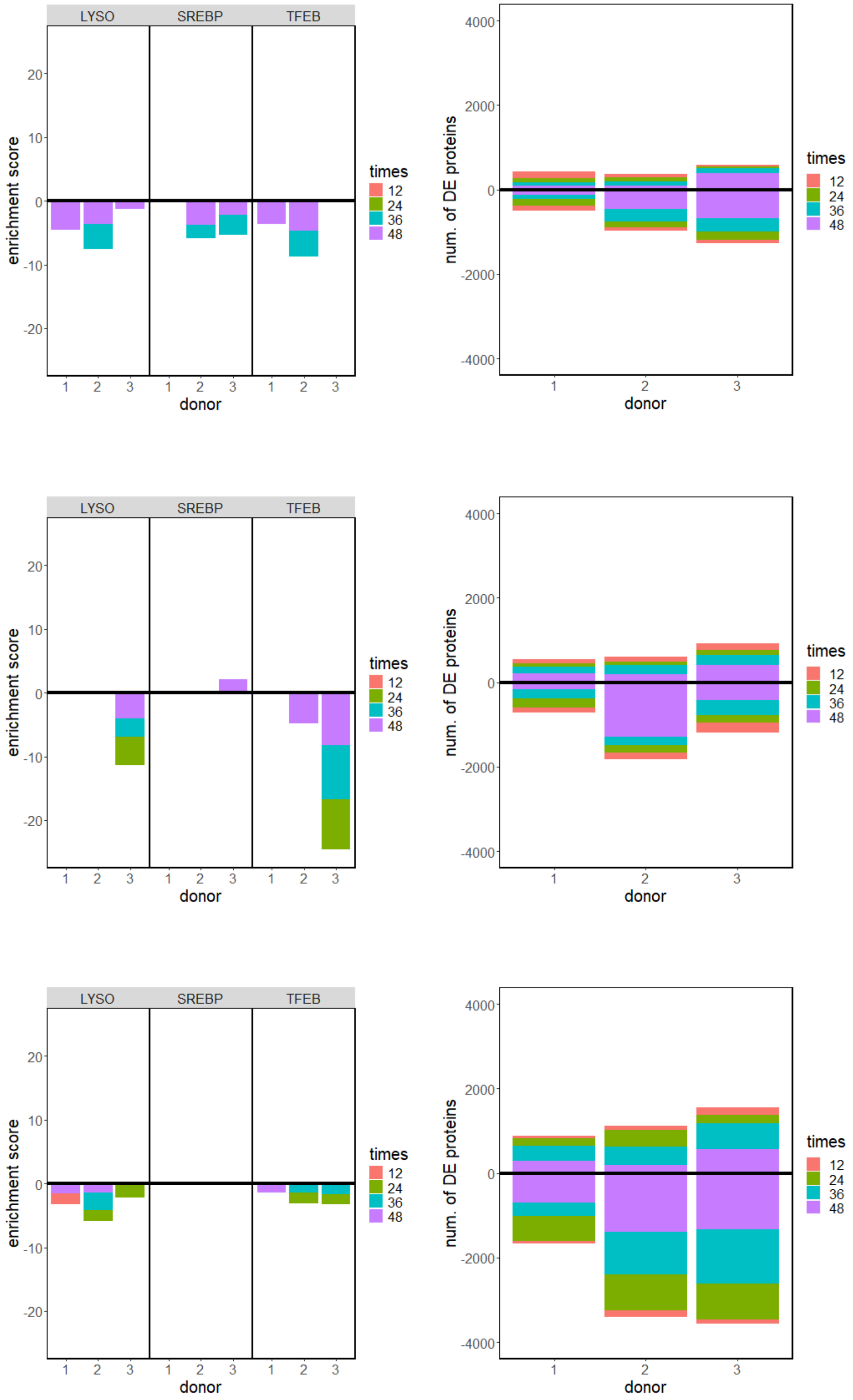

Supplement Figure 2. Protein expression enrichment in three distinct lipid-related gene sets. Enrichment of three distinct gene sets related to lipid-associated activity (lysosome proteins, SREBP-responsive genes, and TFEB-responsive genes) was determined for differentially expressed (both increased and decreased expression) proteins for each infection condition were identified. The results were plotted according to the degree of significance at each time point (indicated with color) and donor (indicated with x-axis numbers) using the -log10 of the significance p-value, with enrichment among up-regulated genes placed above the line and enrichment among down-regulated genes placed below the line, for each of the three cell types as indicated (left panels). Each individual-colored block in the stacked bar graph indicates the degree of enrichment for significantly changed genes for each individual time point (the colored blocks are stacked into a single bar). The result is that the total height of each multi-colored bar indicates the cumulative total degree of gene set enrichment across all time points for individual donors. To compare the downward trend of lipid-related processes to overall protein expression trends, the number of overall significantly up- and down-regulated proteins was plotted at each time point (indicated with color) and donor (indicated with x-axis numbers) for each of the three cell types as indicated (right panels) with stacked blocks for each time point as in the left panels. Data for fibroblasts (**A** FB), human airway epithelial cells cultures (**B** HAE) and microvascular endothelial cells (**C** MVE) were included in our analysis. LYSO- lysosomal proteins; SREBP- sterol regulatory element binding protein; TFEB- transcription factor EB; FB-fibroblast, MVE- microvascular endothelial cells, HAE-human airway epithelial cells cultures

Supplemental Figure 3

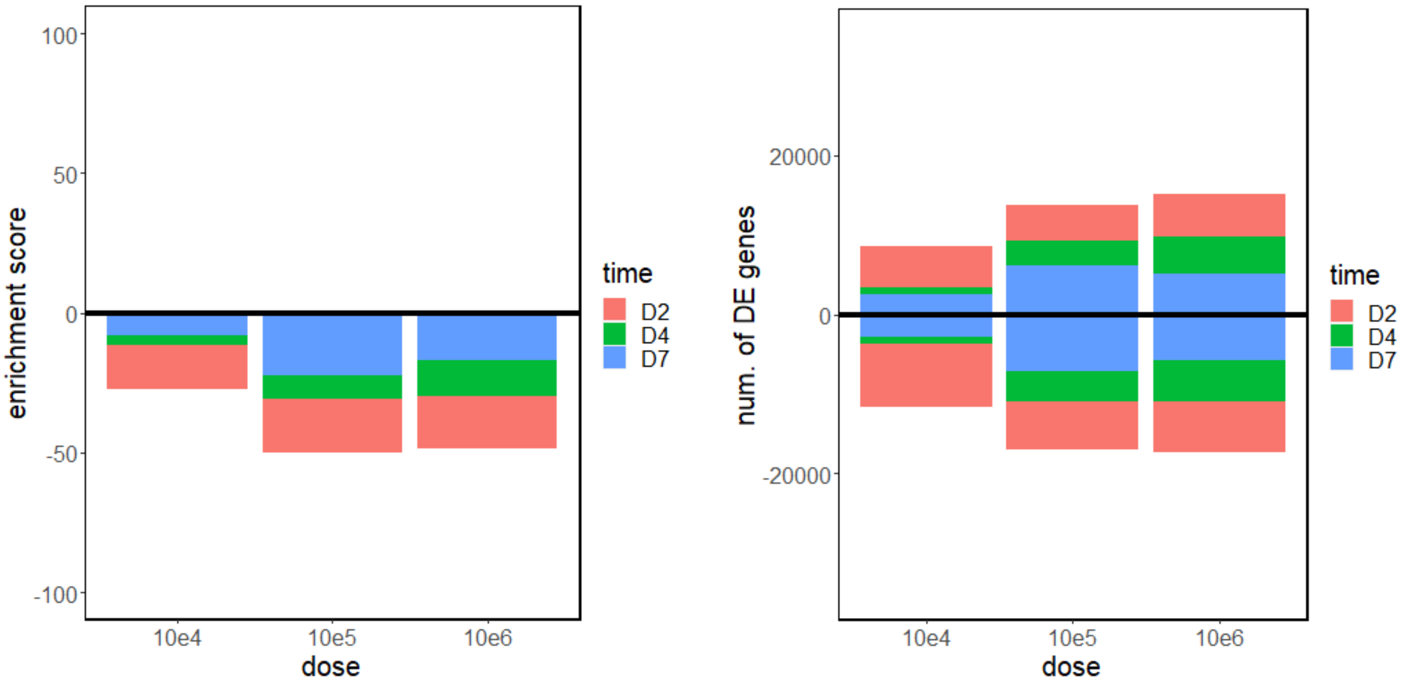

Supplement Figure 3. Protein expression enrichment among lipid-related genes from differentially expressed transcripts from mouse lung. We identified differentially expressed lipid-related protein expression patterns in MERS-CoV infected mouse lungs to determine if the results were similar or distinct to our findings in primary human lung cells. As described in detail in Sims, et al. 2025 (17), the inferior left lung lobes from mock-infected and MERS-CoV infected mice were subjected to MPLEx extraction (chloroform/methanol, 2:1 mix (88-90)) resulting in the simultaneous inactivation of replication competent virus and extraction of proteins, lipids, and metabolites. Samples were safety tested and frozen prior to additional processing. Proteomic analysis was performed as previously described (17). Results are displayed as in Supplemental Figures 1 and 2, but in this case, the analysis was performed on the three infection doses used in these experiments, and for a single gene set: the Lipid Maps Gene/Proteome Database. D- day post infection; DE- differentially expressed

Supplemental Figure 4

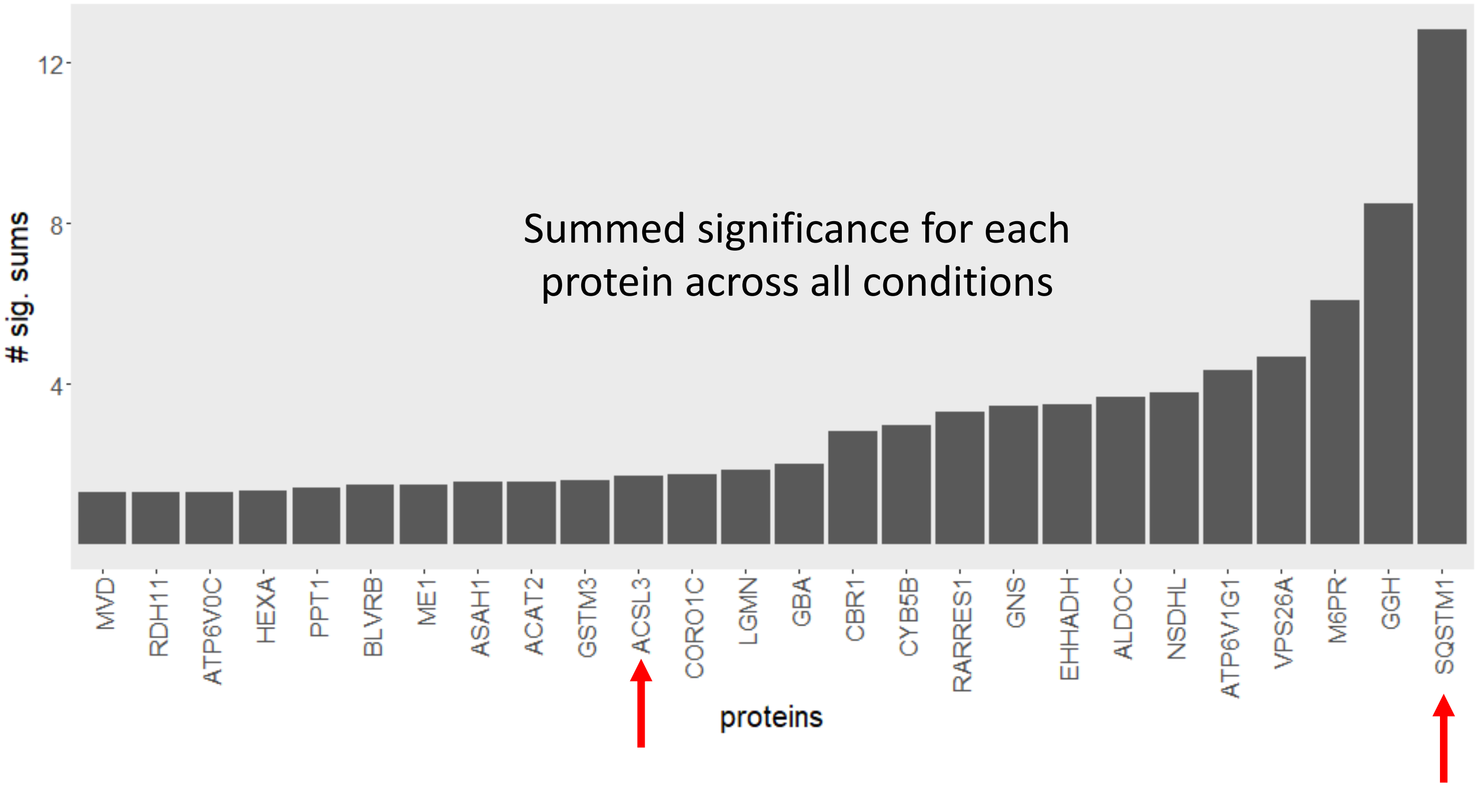

Supplement Figure 4. Summed significance for each lipid-associated protein across all conditions in SARS-CoV infection. The summed significance for each lipid-associated protein across all conditions for SARS-CoV infected epithelial cells was plotted. The sum of all -log10 p-values of significance of up-regulated lipid-associated proteins across all infection conditions is represented. Red arrows highlight proteins of particular interest: ACSL3 and SQSTM1. MVD- mevalonate diphosphate decarboxylase; RDH11- retinol dehydrogenase 11; ATPV0C- ATPase H+ transporting V0 subunit c; HEXA- hexosaminidase subunit alpha; PPT1- palmitoyl-protein thioesterase 1 ; BLVRB- biliverdin reductase B; ME1- malic enzyme 1; ASAH1- acid ceramidase; ACAT2- acetyl-CoA acetyltransferase 2; GSTM3- glutathione S-transferase mu 3; ACSL3- acyl-CoA-synthase long chain family member 3 ; CORO1C- coronin 1C; LGMN- legumain; GBA- glucosylceramidase beta 1; CBR1- carbonyl reductase 1; CYB5B- cytochrome b5 type B; RARRES1- retinoic acid receptor responder 1; GNS- glucosamine (N-acetyl)-6-sulfatase; EHHADH- enoyl-CoA hydratase; ALDOC- aldolase, fructose-bisphosphate C; NSDHL- NAD(P) dependent 3-beta-hydroxysteroid; ATP6V1G1- ATPase H+ transporting subunit G1; VPS26A- VPS2 retromer complex component A; M6PR- mannose-6-phosphate receptor; GGH-gamma-glutamyl hydrolase; SQSTM1- sequestosome 1

Supplemental Figure 5

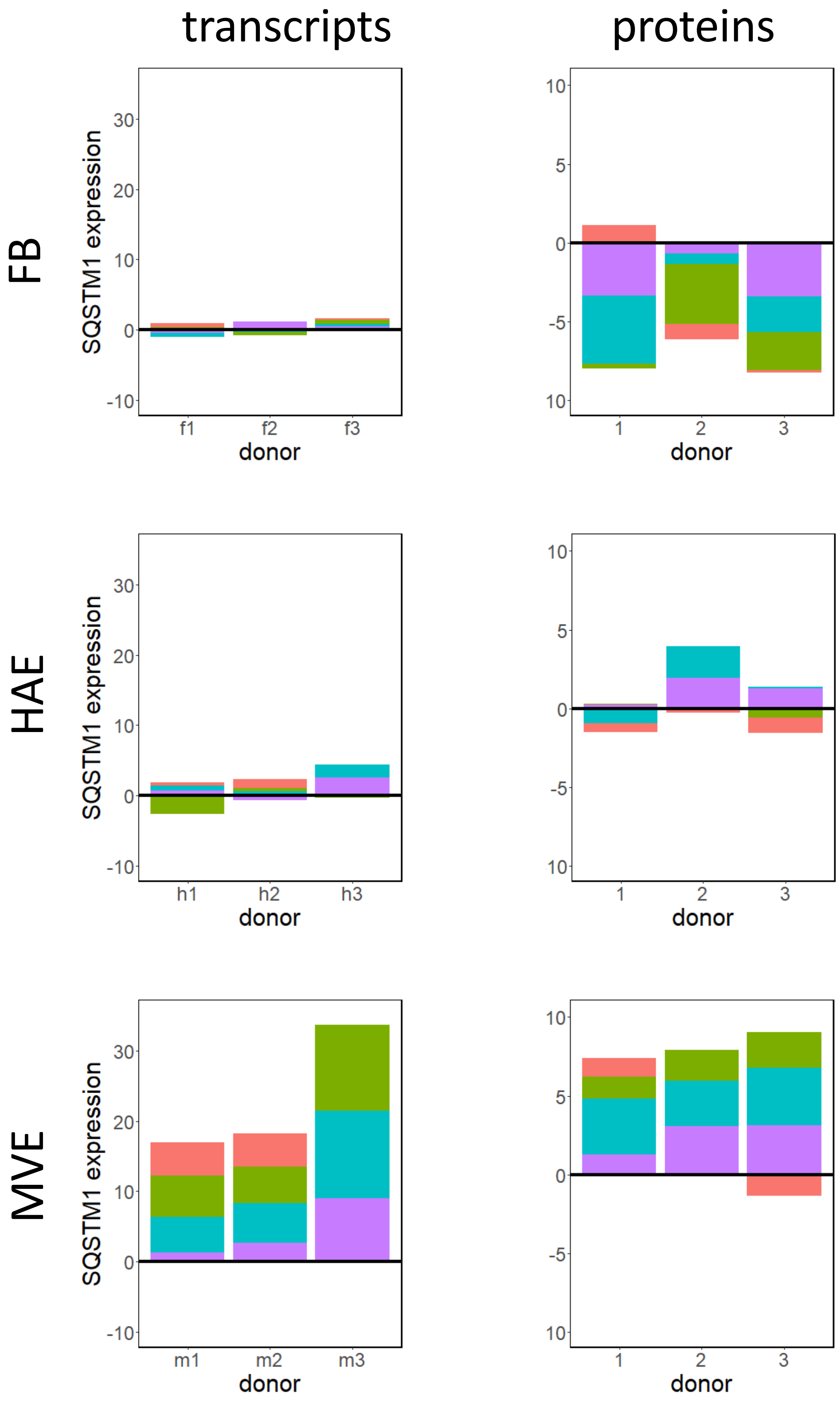

Supplement Figure 5 Differential expression of SQSTM1 transcripts and proteins. SQSTM1 transcript (left panels) and protein (right panels) levels were plotted according to the degree of differential expression at each time point (indicated with color) using the -log10 of the significance p-value, with up-regulation placed above the line and down-regulation placed below the line. Each individual-colored block in the stacked bar graph indicates the significance level for SQSTM1 gene/protein for each individual time point (the colored blocks are stacked into a single bar, rather than being individual bars that each extend from/to the zero line). SQSTM1-sequestosome 1; FB or f-fibroblast; MVE or m-microvascular endothelial cells; HAE or h-human airway epithelial cells cultures

Supplemental Figure 6

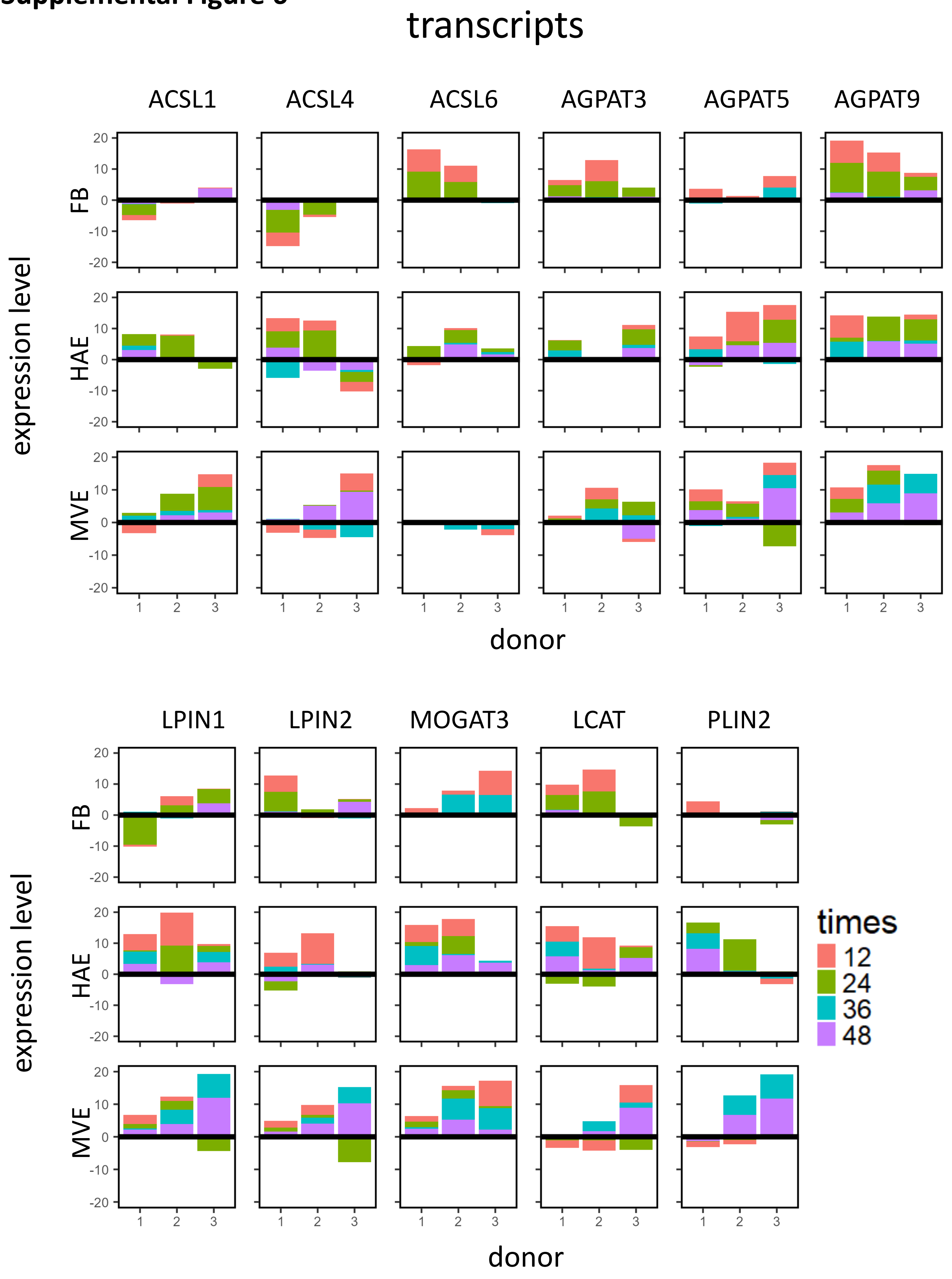

Supplement Figure 6 Transcript expression levels of lipid synthesis/marker genes. To fully explore how MERS-CoV infection alters expression of genes involved in the synthesis of TG and cholesterol esters (both are important components of LDs) we plotted differential transcript expression for genes other than ACSL3 that are involved in TG and cholesterol ester synthesis pathways. Plots are displayed using the same approach as in Figure 4. All 6 genes on the top row and the first 3 of the second row are enzymes involved in TG synthesis. LCAT is involved in cholesterol ester synthesis, while PLIN2 is a protein marker for lipid droplets. Only relevant genes showing some level of significant up-regulation are displayed. Each individual-colored block in the stacked bar graphs indicates the significance level for each gene for each individual time point (the colored blocks are stacked into a single bar, rather than being individual bars that each extend from/to the zero line). ACSL1-acyl-CoA synthetase long chain family member 1; ACSL4- acyl-CoA synthetase long chain family member 4; ACSL6- acyl-CoA synthetase long chain family member 6; AGPAT3- 1-acylglycerol-3-phosphate O-acyltransferase 3; AGPAT5-1-acylglycerol-3-phosphate O-acyltransferase 5; AGPAT9- 1-acylglycerol-3-phosphate O-acyltransferase 9; LPIN1-Lipin-1; LPIN2-Lipin-2; MOGAT3-monoacylglycerol O-acyltransferase, LCAT- lecithin cholesterol acyltransferase, PLIN2-perilipin 2 or adipophilin; FB-fibroblast, MVE-microvascular endothelial cells, HAE-human airway epithelial cells cultures

Supplemental Figure 7

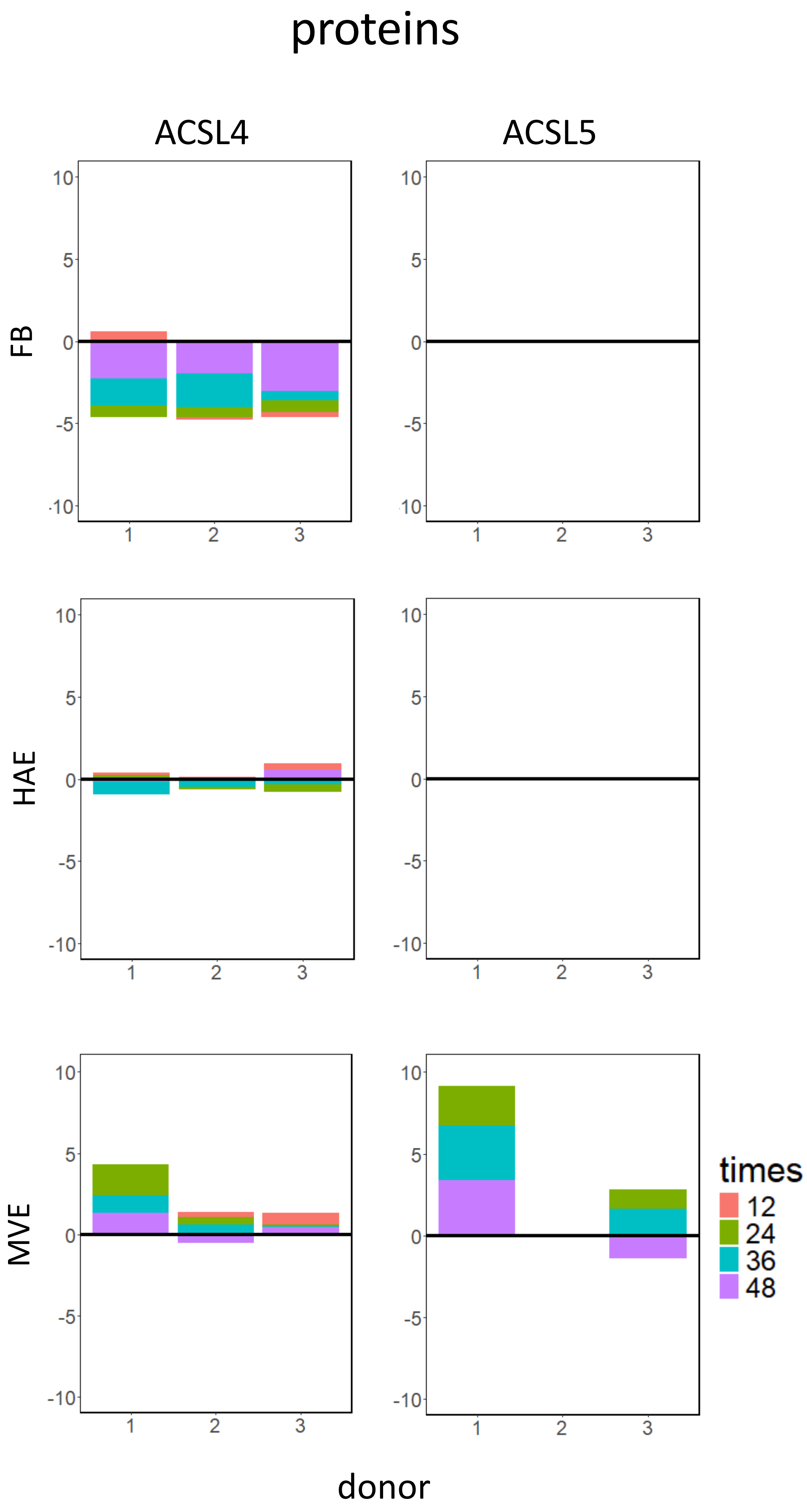

Supplement Figure 7 Protein expression of ACSL4 and ACSL5. As increased protein expression of ACSL3 was detected in MERS-CoV infected primary human lung cells, we also determined the expression levels for the related proteins ACSL4 and ACSL5. Plots are displayed using the same approach as in Figure 4. Of the genes involved in TG or cholesterol ester synthesis, we only detected modest up-regulation in ACSL4 and ACSL5 protein. Each individual-colored block in the stacked bar graphs indicates the significance level for each gene at each individual time point (the colored blocks are stacked into a single bar, rather than being individual bars that each extend from/to the zero line). ACSL4- acyl-CoA synthetase long chain family member 4; ACSL5- acyl-CoA synthetase long chain family member 5; FB-fibroblast, MVE-microvascular endothelial cells, HAE-human airway epithelial cells cultures

Supplemental Figure 8

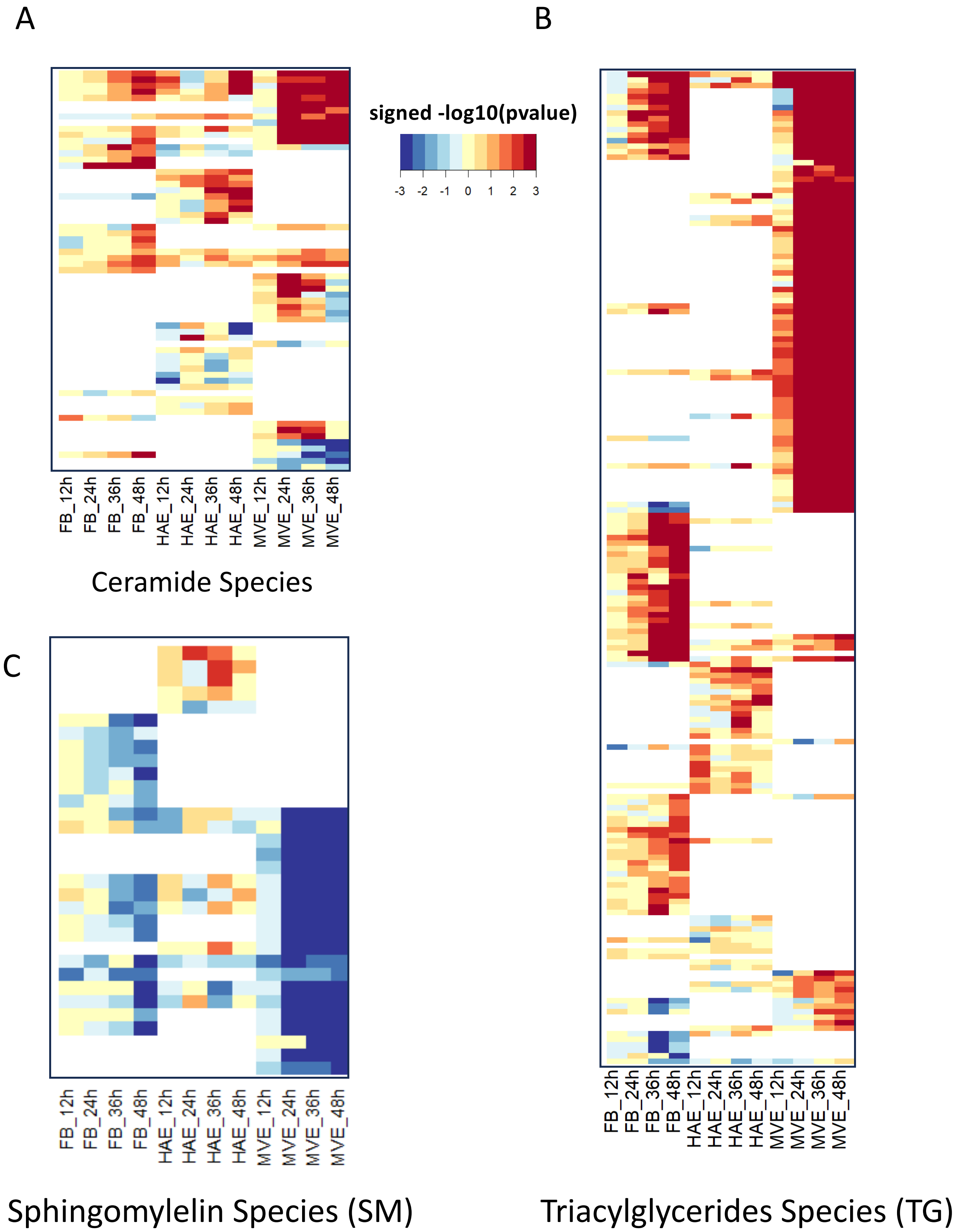

Supplement Figure 8. Expression heatmaps of individual species of three different lipid classes: Heatmaps demonstrating differential lipid species expression above or below mock-infected samples are show for (**A)** ceramide, (**B**) triacylglycerides (TG) and (**C**) sphingomyelin (SM) species. The -log10 of adjusted p-values (signed by the direction of the log2 fold change) from differential abundance tests collapsed among donors using the average of the three values is depicted. FB-fibroblast; MVE-microvascular endothelial cells; HAE-human airway epithelial cells cultures

Supplemental Figure 9

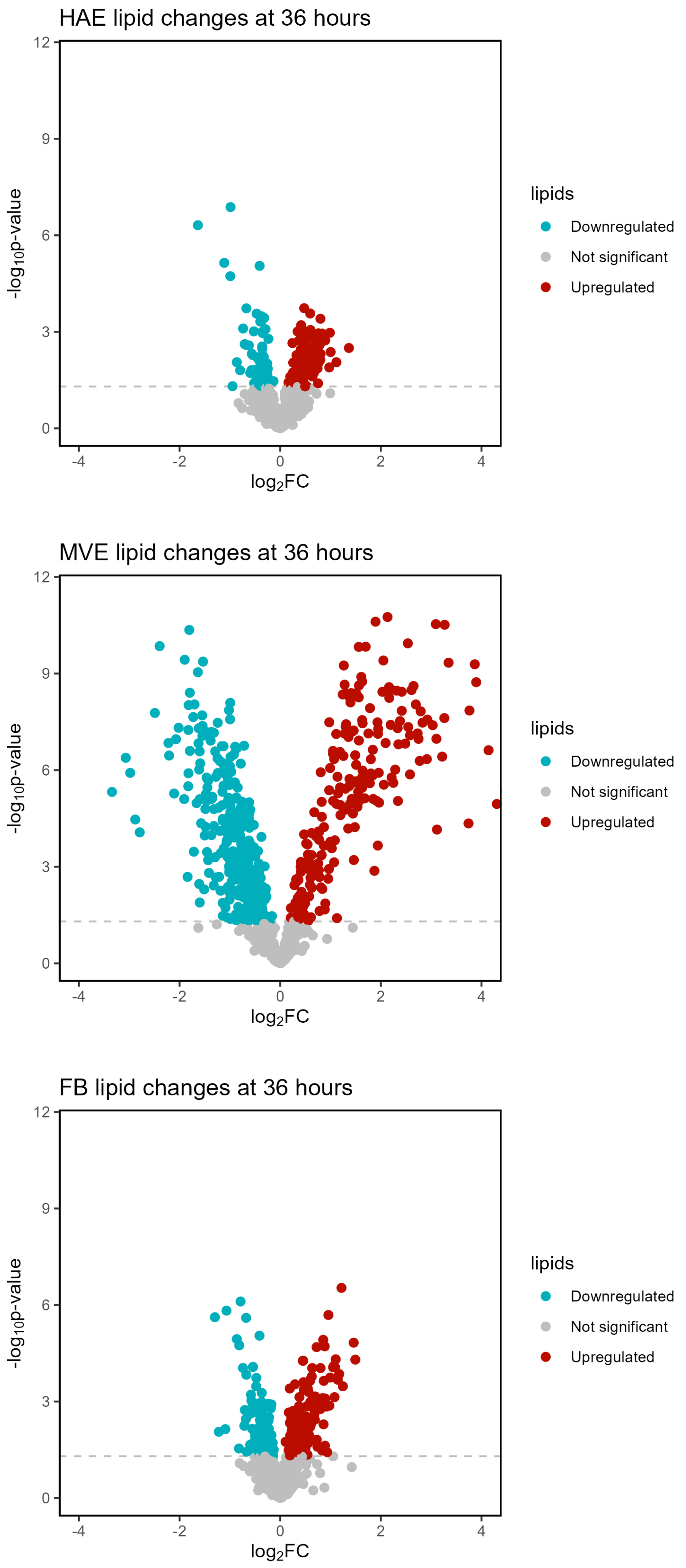

Supplemental Figure 9. Volcano plots of differentially expressed lipids at 36-hours post infection. Fold change and p-values from lipid differential abundance analysis were plotted from the 36-hour time point. Results from individual lipid species in each of the three donors were combined and plotted together for each cell type as indicated. FB-fibroblast; MVE-microvascular endothelial cells; HAE-human airway epithelial cells cultures

Supplementary Figure 10

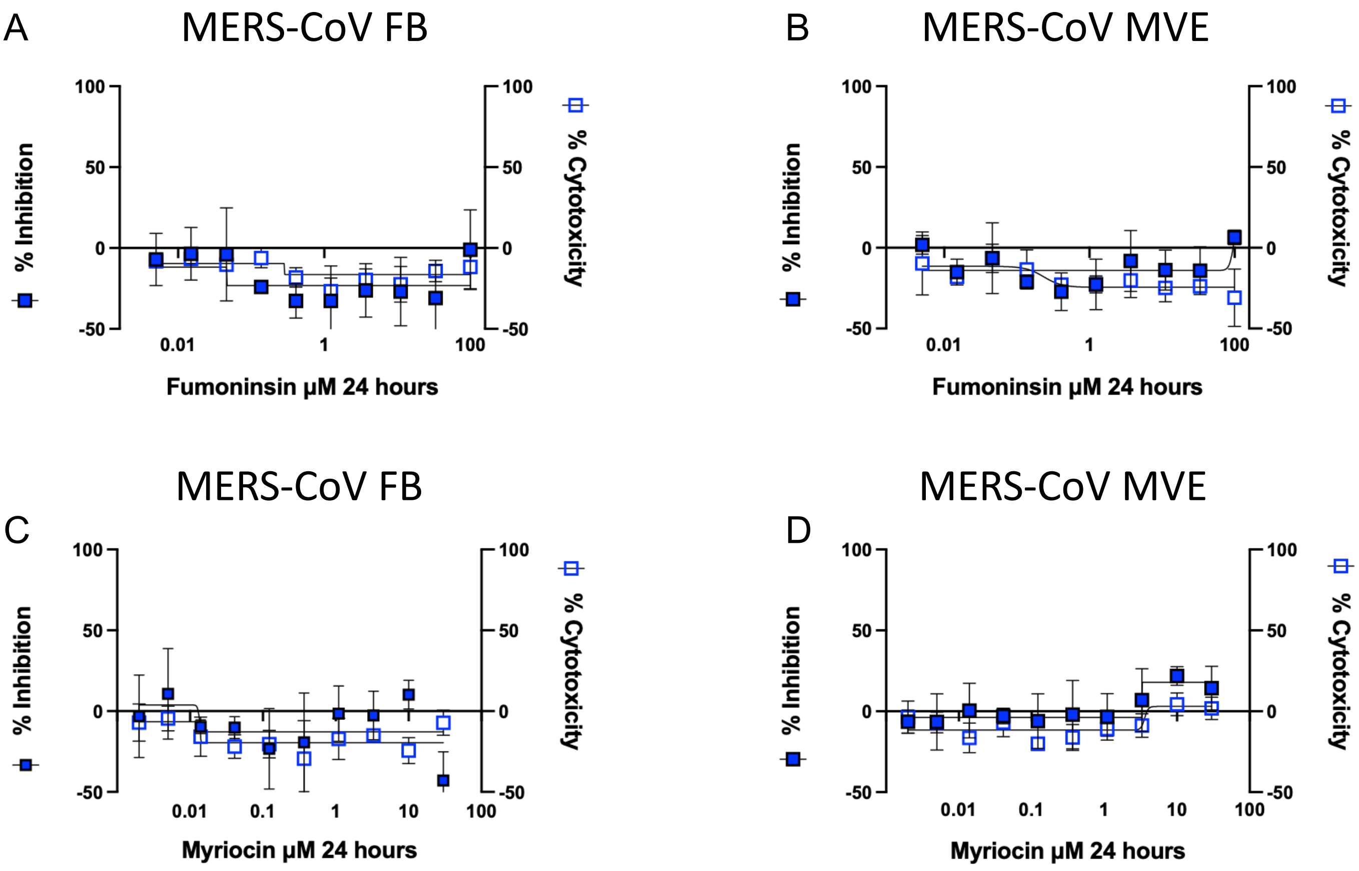

Supplement Figure 10. Effects of de novo ceramide synthesis inhibitors on MERS-CoV replication. Fumonisin B1 (**A-B**) and Myriocin (**C-D**) were used to treat primary human lung fibroblasts (**A, C**) or microvascular endothelial cells (**B, D**) for 24 hours during MERS-CoV infection. A range of doses were used to test drug toxicity in uninfected cells (as assessed by ATP levels, unfilled symbols), and virus replication (as assessed by luciferase production, filled symbols) as shown. FB-fibroblast; MVE-microvascular endothelial cells; MERS-CoV- Middle East respiratory syndrome coronavirus

Supplemental Figure 11

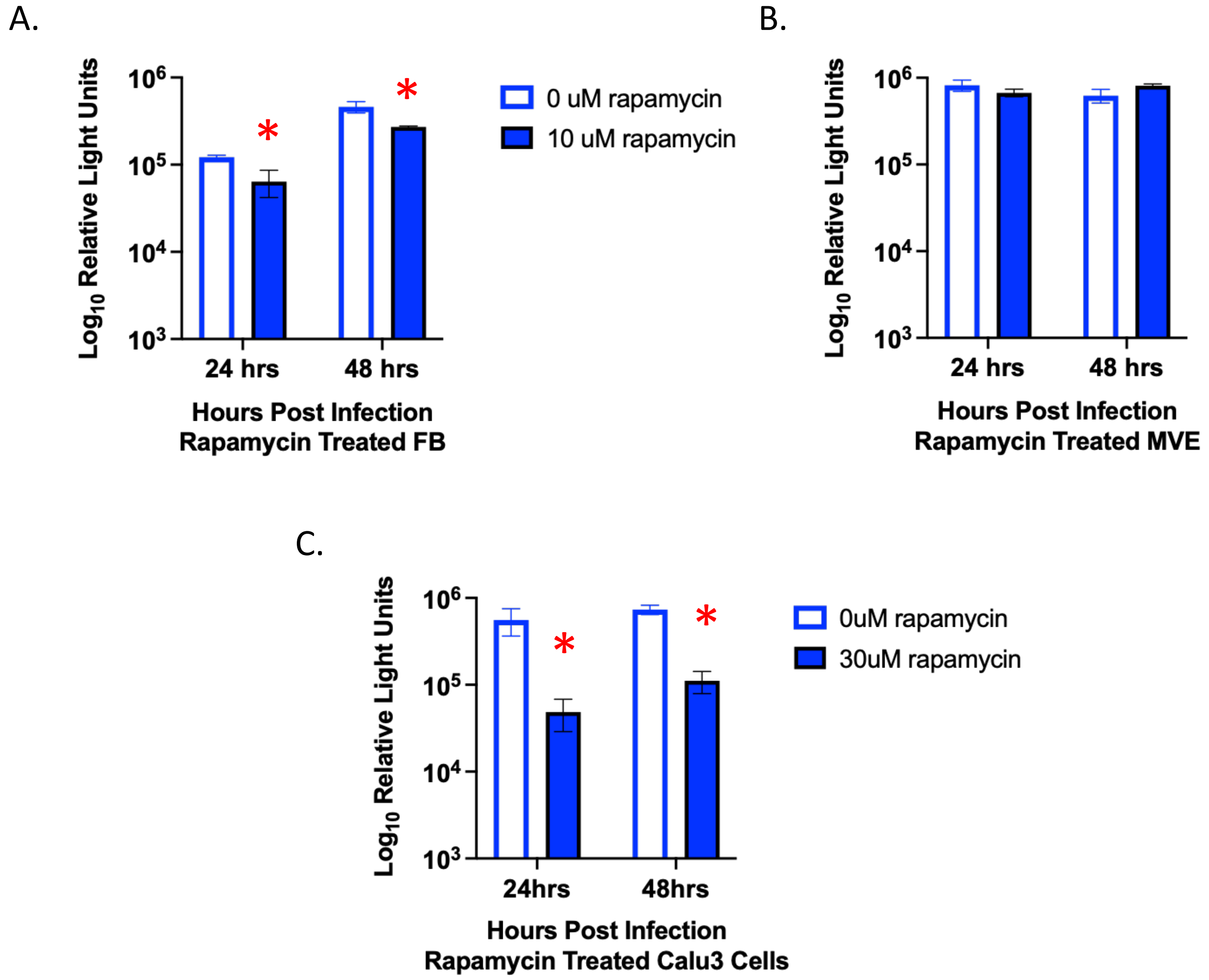

Supplement Figure 11. Effect of rapamycin treatment on MERS-CoV replication.

Rapamycin treatment of FB (**A**), MVE (**B**) and Calu3 (**C**) cells for 24- and 48-hours during MERS-LUC infection. Single dose rapamycin treatment 10 uM (**A-B**) or 30 uM (**C**) was used to assess whether levels of luciferase expression as a surrogate for MERS-CoV replication (filled symbols) were changed in the presence or absence of inhibitor. FB-fibroblast; MVE-microvascular endothelial cells; hrs- hours post infection
